# Facilitators, Barriers, and Cultural Appropriateness of Mindfulness-Based Interventions Among Saudi Female University Students: Qualitative Study

**DOI:** 10.2196/78532

**Published:** 2025-12-19

**Authors:** Duaa H Alrashdi, Carly Meyer, Rebecca L Gould

**Affiliations:** 1 Division of Psychiatry, Faculty of Brain Sciences, University College London, Maple House, 4th Fl, Tottenham Ct Rd, London, W1T 7NF, United Kingdom, 44 7867307272; 2 Department of Health Sciences, Faculty of Health and Rehabilitation Sciences, Princess Nourah Bint Abdulrahman University, Riyadh, Saudi Arabia; 3 Bolton Clarke Research Institute, Brisbane, Australia; 4 Department of Clinical, Educational, and Health Psychology, Faculty of Brain Sciences, University College London, London, United Kingdom

**Keywords:** mindfulness, university student, Saudi Arabia, mental health, qualitative study, culture

## Abstract

**Background:**

Mindfulness-based interventions (MBIs) have been shown to improve university students’ well-being. However, previous studies have not systematically explored factors that can facilitate or hinder engagement in MBIs among Saudi university students, nor how MBIs can be culturally adapted to meet their needs.

**Objective:**

This study aimed to (1) explore the perspectives of Saudi female university students about factors influencing engagement with MBIs, (2) explore the cultural appropriateness of MBIs, and (3) systematically identify recommendations for developing a culturally appropriate MBI.

**Methods:**

A qualitative research approach was used to collect data using semistructured individual interviews and focus groups. Two established frameworks for behavioral interventions were applied to guide the interview topics and data analysis. The COM-B (Capability, Opportunity, and Motivation Domains of Behavior Change) model was applied to identify potential enablers and barriers influencing students’ engagement with MBIs. The cultural adaptation framework by Bernal et al was used to explore the cultural appropriateness of MBIs. Subsequently, recommendations for developing MBIs, with a specific focus on an online version, were systematically formulated using the Theory and Techniques Tool. Data were analyzed using mixed inductive-deductive thematic analysis.

**Results:**

Fourteen Saudi female university students (mean age 24, SD 4.9 years) participated in semistructured interviews and focus groups. Numerous potential enablers and barriers to MBI engagement were identified. Factors that may influence engagement pertained to capability (variation in knowledge of mindfulness), opportunity (anticipated difficulty finding time), and motivation (variation in anticipated and experienced benefits of mindfulness). Participants also highlighted several considerations that may enhance the cultural relevance of MBIs, drawing on the cultural adaptation domains by Bernal et al. These included the importance of aligning MBIs with the local cultural context, incorporating metaphors and examples rooted in Saudi and Arab culture, and accommodating students’ preferences for the duration of MBIs. Key recommendations for developing culturally appropriate MBIs for Saudi university students included providing clear information to improve understanding of mindfulness, providing practical strategies and skills to overcome barriers such as time constraints, delivering MBIs in both Arabic and English, and ensuring that MBIs’ content aligns with local cultural values and contexts.

**Conclusions:**

Findings and recommendations aim to enhance the feasibility, acceptability, engagement, and effectiveness of MBIs among Saudi university students, particularly female students. However, whether they do in fact achieve these aims is unknown. Future research should endeavor to evaluate the effectiveness of proposed recommendations and explore the enablers and barriers to MBI engagement in a broader population of Saudi students.

## Introduction

Mindfulness-based interventions (MBIs) are interventions that are grounded in the practice of mindfulness, typically defined as the “awareness cultivated by paying attention in a sustained and practical way: on purpose, in the present moment, and non-judgmentally” [1]. Examples of MBIs include mindfulness-based cognitive therapy and mindfulness-based stress reduction. Both online and face-to-face MBIs have been shown to improve the mental health and well-being of university students, including reducing depression, stress, and anxiety [2,3]. Yet despite the effectiveness of MBIs, previous reviews indicate that many students who embark on MBIs do not complete them. For example, a meta-analysis of 23 studies of online MBIs reported an overall attrition rate of 34% [2]. The reasons underlying this are not clearly understood. However, some have argued they might include a perceived lack of time and requiring too much effort, along with the challenge of adopting new habits for regular practice among university students [4].

Improving MBI design to facilitate student engagement is one way of potentially increasing MBI completion levels. Recent studies have involved university students in the development of MBIs to ensure that interventions are more relevant and responsive to students’ needs and preferences [5,6]. However, no studies, to date, have systematically explored potential barriers to and facilitators of intervention engagement in university students using an established theory-informed approach to inform MBI design. Therefore, this study adopted a behavioral science lens to better understand students’ engagement with MBIs. The COM-B (Capability, Opportunity, and Motivation Domains of Behavior Change) framework suggests that to implement a successful behavioral intervention (such as an MBI), a comprehensive analysis of the underlying behavioral determinants is needed [7]. Such an analysis requires consideration of 3 essential domains: capability, the physical and psychological abilities of a person to engage in an activity; opportunity, factors within both the physical and social environment that lie beyond the person and can either facilitate or hinder activities; and motivation, the internal mental processes within the person that can influence engagement in activities and includes both reflective and automatic processes [7]. The COM-B framework is widely used in health care settings, including the development of mental health interventions [8,9], and was therefore applied in this study to guide the development of interview topic guides and data analysis.

It is a commonly accepted practice to align COM-B findings with the Theoretical Domains Framework (TDF) [10-12] to identify strategies to improve intervention development. The TDF is a comprehensive synthesis of psychological theories and constructs, which includes domains that are used to understand factors influencing behavioral change [10]. These domains represent the theoretical constructs mediating an intervention’s effects [13], which should align with anticipated barriers and enablers. The TDF can be considered an extension of the COM-B model, as it provides a more detailed framework for exploring behavioral determinants [12]. In this study, it was used to expand on the COM-B findings by mapping the identified factors that may influence students’ engagement with MBIs onto relevant theoretical constructs.

Furthermore, the Theory and Techniques Tool is an interactive mapping resource designed to identify links between the theoretical constructs of the TDF and relevant behavior change techniques (BCTs), developed based on a consensus process [13-15]. This tool enables the selection of BCTs that directly target identified barriers and enablers, thereby informing the design of tailored interventions [13,14]. The TDF, as noted earlier, is used to identify and understand behavioral determinants, which can subsequently be mapped onto the Theory and Techniques Tool to develop evidence-based recommendations. This approach has previously been applied to the development of health care interventions, such as aiding mental health professionals in delivering behavioral activation interventions to young people, and informing the delivery of pharmacy-based interventions [16,17]. Consequently, the Theory and Techniques Tool was used in this study to guide the development of recommendations for improving engagement with MBIs.

In addition to examining barriers to and facilitators of intervention engagement, it is crucial to consider the cultural appropriateness of interventions for the target population to improve both their effectiveness and retention levels [18]. A recent systematic review identified only two MBI studies conducted in the Kingdom of Saudi Arabia (KSA), both involving Saudi university students [19]. One small randomized controlled trial (RCT) included 40 male students and reported a statistically significant improvement in psychological distress among those who received an MBI compared with a no-treatment control at postintervention [20]. In contrast, another pilot RCT included 26 female students and found no statistically significant differences in psychological well-being, life satisfaction, mindfulness traits, depression, or stress symptoms between the MBI group and an active control at postintervention [21]. Although both studies implemented some degree of cultural adaptation, for example, by modifying intervention delivery to better align with the target population, and with one study further adapting the intervention content (eg, incorporating elements of Islamic spirituality) [21], they generally fell short of achieving comprehensive cultural adaptation [19]. These findings have raised questions about the extent to which these studies met key cultural considerations (eg, whether the interventions were conceptually grounded in, or goal-oriented toward, alignment with participants’ cultural backgrounds) that are believed to influence the effectiveness of interventions implemented outside their original cultural context [19]. Critical methodological limitations were evident in both studies, including high or unreported attrition rates and small sample sizes that were underpowered to examine effectiveness or detect significant between-group differences. Notably, neither study conducted a formal analysis of participants’ experiences with the interventions, which may have resulted in a lack of clarity regarding students’ perceptions and the overall acceptability of such interventions. A similar lack of culturally adapted MBIs has been observed across the broader Gulf Cooperation Council region, which shares similar cultural and regulatory contexts with KSA [19]. It is worth noting that although the studies conducted in this region demonstrated a limited degree of cultural adaptation (eg, adjustments to intervention delivery were made) [19], none developed their intervention protocols using systematic or theory-driven frameworks to address cultural considerations or enhance participant engagement, which may have limited the robustness and cultural relevance of these interventions. This gap highlighted the need to explore culturally sensitive, theory-informed MBIs tailored to Saudi populations.

One way to systematically develop culturally appropriate MBIs for Saudi university students is to use an established theory-informed approach such as the cultural adaptation framework by Bernal et al [18,22]. This framework has previously been used to evaluate the cultural appropriateness of mental health interventions [19,23,24]. It considers cultural modifications with respect to eight domains: (1) language (eg, the language of MBI materials), (2) people (eg, the gender of instructors delivering mindfulness materials and how written language is presented), (3) metaphor (eg, the use of symbols, sayings, wisdom, poems, and similar that resonate culturally with students), (4) content (eg, students’ perspectives about the core components typically included in MBIs), (5) goal (eg, potential goals that students aim to achieve through participation in online MBIs), (6) concept (eg, how students conceptualize mindfulness and how it can be presented to them in an acceptable manner), (7) method (eg, the practical procedures of delivering online MBIs from a student’s perspective), and (8) context (eg, the general integration of MBIs, including online versions, within the culture and university setting).

At present, it remains challenging to draw robust conclusions about the acceptability or effectiveness of MBIs among Saudi university students, given the limited number of studies conducted in the KSA, including one pilot RCT and another small-scale RCT, both with small sample sizes that were underpowered to examine effectiveness, and, more broadly, in comparable populations such as those in the Gulf Cooperation Region. Although this scarcity of evidence applies to both female and male Saudi university students, this study focused on Saudi female students, based on previous findings indicating that being female is associated with a higher prevalence of mental health challenges among Saudi university students [25,26]. For instance, symptoms of depression have been reported to be significantly more prevalent among females (50%) than males (39%) [26]. Such findings have prompted calls for initiatives specifically tailored to address Saudi women’s mental health [27]. Additionally, while this study explored MBIs in general, it placed particular emphasis on online delivery. It has been suggested that self-guided MBIs (eg, online versions) can be cost-effective, particularly in contexts where large numbers of users can access them [28]. Furthermore, online MBIs have been proven to effectively improve students’ psychological well-being [2], suggesting they are a viable option for increasing accessibility for this population.

Consequently, given the lack of knowledge about factors that can facilitate or hinder engagement in MBIs among university students, and how MBIs can be culturally adapted to meet the needs of Saudi female students, this study aimed to (1) explore potential barriers to and facilitators of engagement in MBIs in a Saudi context using the COM-B framework; (2) explore Saudi female university students’ perceptions of mindfulness and how to culturally adapt MBIs, particularly online interventions, using the cultural adaptation framework by Bernal et al [22]; and (3) systematically identify a series of recommendations using Theory and Techniques Tool that could be incorporated into the development of an MBI for Saudi university students, especially female students, in order to potentially enhance its effectiveness.

## Methods

### Study Design

A qualitative research approach was used to collect data in this study using semistructured individual interviews and focus groups with Saudi female university students. This study was reported in line with the Consolidated Criteria for Reporting Qualitative Research (COREQ) checklist [29].

### Materials

The interview topic guides were developed based on two frameworks (see Multimedia Appendix 1). The COM-B framework [7] was used to understand factors influencing engagement with MBIs (eg, Do you have any physical health conditions that could impact your ability to practice mindfulness or meditation exercises? How would you juggle practicing online mindfulness or meditation exercises alongside other demands on your time?). The cultural adaptation framework by Bernal et al [18,22] was used to address the cultural appropriateness of MBIs, including online versions (eg, What kind of metaphors or sayings do you think we should include in an online MBI? What do you hope an online MBI could be used for?). Prompts, including examples of mindfulness exercises, definitions, and concepts, were presented to all participants and used throughout the interviews to explore students’ perspectives in more depth.

### Participants

Participants were recruited from a women-only government university in KSA. Inclusion criteria were as follows: (1) currently enrolled at the university as a student, (2) aged 18 years and over, (3) fluent in Arabic language, and (4) has access to the internet. There were no specific exclusion criteria. As this study was exploratory and aimed to gather initial data, particularly given the limited research in KSA, participants were not purposively selected or allocated to focus groups based on their prior mindfulness experience. Information on participants’ prior mindfulness experience was collected solely to provide background context, and so the same topic guide was used for all participants.

A cohort of 12 participants has been suggested to be sufficient for gathering in-depth information from homogenous populations [30]. Therefore, we sought to recruit 12‐15 participants, as this study focused on a homogeneous group of Saudi female university students and aimed to collect in-depth information. Additionally, the dataset collected was substantial, as it was guided by two theoretical frameworks, and so given the focused and detailed nature of this study, this range was considered sufficient to generate meaningful insights aligned with the aims of this in-depth exploration. Participant recruitment continued until the target range was reached, and no new themes were observed to be generated. This cohort therefore has the potential to be transferable to other female sectors of universities within the KSA, as these institutions typically share a similar gender-segregated education system.

### Ethical Considerations

Ethical approval was granted from both the research ethics committee at University College London (23503/001) and the research ethics committee at the government university in KSA (22‐0390). This study was conducted in accordance with Helsinki Declaration guidelines. Informed consent was obtained from all participants prior to their inclusion in the study. All participant data were stored in password-protected files and pseudonymized to ensure the privacy and autonomy of participants. No compensation was provided to participants.

### Procedure

Participant recruitment and interviews were conducted from November 2022 to January 2023. Potential participants were informed about the study using flyers and a brief study description that were disseminated via email and messaging platforms, and via academic staff presentations to their respective students. Potential participants expressing interest were invited to contact the first author (DHA) and were provided with an information sheet and asked to confirm their eligibility using an online survey (ie, Qualtrics). Fully informed consent was subsequently collected from all participants. Those who consented were asked to provide demographic information (eg, age), as well as their preferred email address and meeting time, also collected via Qualtrics. Reminders were sent to students who had registered their interest but had not consented to the study approximately 1 week after their initial contact.

All participants were invited to participate in a focus group; those who were unable to attend a focus group were interviewed individually. Individual interviews and focus groups were conducted via videoconferencing software (ie, Microsoft Teams). Participants were asked to turn off their cameras and were encouraged to use fictitious names to maintain their privacy. All interviews were conducted in Arabic by the first author (DHA) and were audio-recorded and auto-transcribed via Microsoft Teams. The first author (DHA) subsequently checked the transcripts for accuracy and removed all identifying information. DHA translated the quotes from Arabic to English for discussion with other research team members (CM and RLG) and for reporting findings. To assess the reliability of the translations, 20% (61/307) of the total coded English quotations were randomly selected across all themes. Two bilingual reviewers (fluent in Arabic and English) independently evaluated these for semantic equivalence with the Arabic originals. Interrater agreement between the reviewers was 95%, and minor revisions were subsequently made to translated quotations to ensure linguistic accuracy.

### Data Analysis

Descriptive statistics were used to describe participants’ characteristics. The dataset was analyzed using mixed inductive-deductive thematic analysis. This approach aligns with the aims of this study as it enables both indexing to the frameworks and inductive generation of themes. First, a “top-down” deductive approach was used, wherein data were extracted in relation to domains and subdomains of the COM-B framework (ie, capability: physical and psychological; opportunity: physical and social; and motivation: reflective and automatic). Data were also extracted in relation to the cultural adaptation framework by Bernal et al [22] (ie, language, people, content, metaphor, method, concept, goal, and context). To facilitate the management of this process, a method similar to the 2013 framework analysis approach by Gale et al [31] was used, in which separate tables for each subdomain and domain were created using Microsoft Word, and all relevant quotations from each participant were referenced to the appropriate table.

Second, an inductive “bottom-up” approach using reflexive thematic analysis [32] was used to identify codes and generate themes within each subdomain and domain of both frameworks. Findings were analyzed at both the semantic level, which captures explicit meanings conveyed by participants, and the latent level, which uncovers underlying, implicit meanings [32]. The first author (DHA) developed codes, subthemes, and themes, and two members of the research team (CM and RLG) independently reviewed the analysis. The reviewing process involved multiple rounds of discussion among the research team, followed by joint meetings until the final themes were agreed upon.

**Figure 1.**
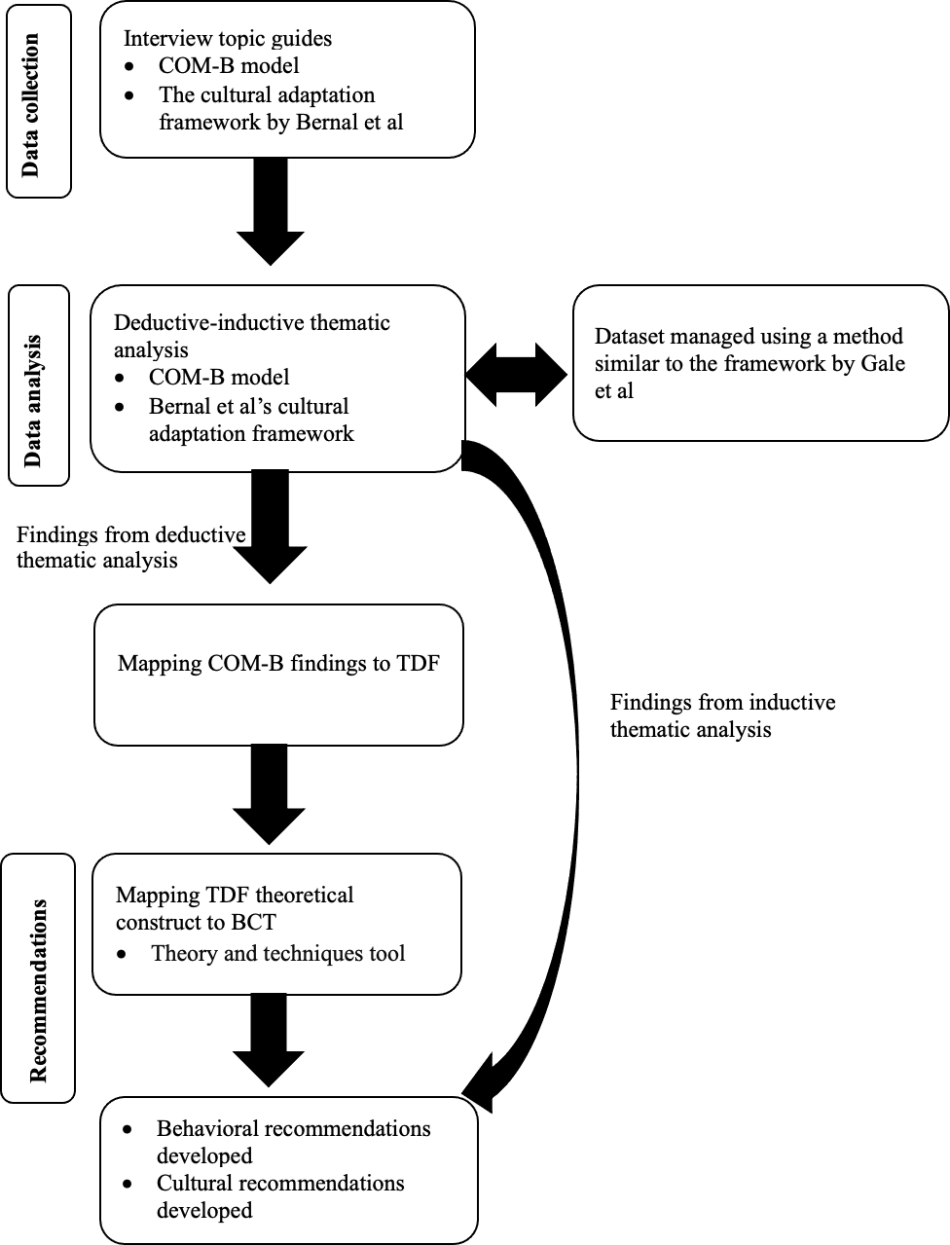
Operationalization of the theoretical frameworks used across data collection, analysis, and recommendation development [22,31]. BCT: behavior change technique; COM-B: Capability, Opportunity, and Motivation Domains of Behavior Change [7]; TDF: Theoretical Domains Framework [10].

A series of recommendations for the development of MBIs, with a specific focus on an online version, was systematically formulated using the Theory and Techniques Tool. Themes within the COM-B domains were mapped to the theoretical constructs of the TDF as per guidance [10]. Subsequently, these theoretical constructs were mapped to BCTs using the online matrix incorporated within the Theory and Techniques Tool, and BCTs with strong consensus were selected [13,14]. Recommendations for MBI development were then formulated based on all selected BCTs, with a few exceptions that were deemed irrelevant (eg, 2.6 Biofeedback, as biofeedback monitoring devices or measurements are not typically used in MBIs). Themes within the cultural adaptation domains by Bernal et al [22] were used to guide the development of culturally specific recommendations, as this framework comprises many practical procedures facilitating its implementation. For example, if a language need was identified, recommendations may include translating and adapting intervention materials into the students’ primary language. DHA developed the recommendations, and CM and RLG independently reviewed them, followed by discussions in several team meetings until they were agreed upon. Figure 1 illustrates how the theoretical frameworks were operationalized throughout the study.

### Reflexivity

DHA, a Saudi female PhD candidate, conducted the interviews after receiving training in qualitative research methodology. DHA had previous experience with MBIs, including participating in an online MBI and conducting research on MBIs with consideration of cultural context. This experience may have fostered a favorable attitude toward the efficacy and benefits of MBIs, as well as personal insights into potential cultural adaptations. Such views might have shaped the way DHA conducted interviews and facilitated focus groups, potentially influencing the questions asked or the interpretation of participant responses. DHA had an unexpected prior relationship with 3 of the participants, who were her classmates in a previous course. However, DHA had no prior relationship with the majority of participants (11/14). Steps were taken to establish a good rapport with all participants during the interviews. DHA maintained a reflexive journal throughout the analysis to document her assumptions and interpretations, ensuring that these were critically examined when necessary. She also engaged in regular debriefing with the research team (CM and RLG) to discuss and refine the findings. Prior to the interviews, participants were informed about the aims and reasons for the study through the informed consent process, which reflected the researchers’ interests. The coauthors have experience in qualitative research and in applying the COM-B framework, TDF, and the Theory and Techniques Tool.

## Results

### Participant Characteristics

A total of 34 students contacted the researcher (DHA). Out of these students, 18 did not provide informed consent and 16 consented. Two students, among those who consented, either did not provide a time for a meeting or did not attend the allocated meeting. In total, 14 students were interviewed, with 4 focus groups being conducted with 10 students (2‐3 students per group), and 4 students being interviewed individually. The average duration of the individual interviews was 1.16 (range 1.06‐1.27) hours, and for the focus groups, it was 1.36 (range 1.27‐1.45) hours.

Table 1 shows participants’ characteristics (N=14). All participants were female Saudi students with an average age of 24 (SD 4.9, range 19‐32) years, and the majority reported being single (n=9, 64%). Half of the participants (n=7, 50%) were enrolled in a bachelor’s program, while the remaining half were enrolled in a master’s program. More than half of the participants (n=8, 57%) reported prior experience with mindfulness, and out of those, 4 (50%) were actively practicing mindfulness at that time, with an average practice time of approximately 11 (SD 11.8, range 2‐28) hours per week.

**Table 1.** Participant characteristics (N=14).

Characteristics	Values, n (%)
Gender	
Female	14 (100)
Age in years	
19‐20	5 (36)
21‐29	7 (50)
30‐32	2 (14)
Nationality	
Saudi	14 (100)
Experience with mindfulness	
Yes	8 (57)
No	6 (43)
Currently practicing mindfulness	4 (22)
Average practice hours per week	
2‐4 hours	2 (50)
10‐28 hours	2 (50)

### Mindfulness Experiences

There were a diverse range of responses in relation to students’ past or current experiences with mindfulness. Although 8 students reported previous experience with mindfulness when providing background information, only 5 could be categorized as having formal mindfulness experience during later questioning. These latter students had learned, practiced, or maintained their mindfulness practice through formal courses or as part of their education, such as programs focused on mindfulness or similar approaches containing mindfulness elements (eg, acceptance and commitment therapy). The remaining 3 students reported that they had become aware of mindfulness only through other sources, such as booklets or social media, and had not completed a formal course but had instead attempted to practice mindfulness on their own. Others indicated that they had neither practiced mindfulness nor read about it. Therefore, subgroup analyses between students with and without formal mindfulness experience were not conducted in this study, as only a small number of participants (5/14) demonstrated sufficient familiarity with mindfulness to permit meaningful comparison.

### The COM-B Framework

#### Overview

Facilitators of and barriers to students’ engagement with MBIs, with a particular focus on online versions, were explored via the COM-B framework. Table 2 shows the COM-B domains and subdomains alongside the key themes and relevant quotations. All themes, subthemes, and illustrative quotations are provided in Multimedia Appendix 2.

**Table 2.** Key findings of the COM-B (Capability, Opportunity, and Motivation Domains of Behavior Change) framework.^a^

Domains, subdomains, and themes	Quotes
Capability	
Physical capability	
No significant health issues impacting mindfulness practice or online course use	“I had [a physical injury] that prevented me...” [Participant M]
Psychological capability	
Variability in knowledge of mindfulness	“Maybe it makes you getting busy from the reality, living more inside, in your thoughts [...]” [Participant A] “...There may be specific groups of people who, for example, have high anxiety, high levels of worry, or those who have obsessions.” [Participant J]
Varied cognitive skills needed to engage in mindfulness practice	“I don’t think it is easy [to pay attention for a specific time], especially for people who easily get distracted...” [Participant A] “I can divide the time and allocate some for it, just like when I allocate time for studying, prayer, and self-care. I can incorporate it into my schedule.” [Participant O]
Opportunity	
Physical opportunity	
Variation in access to appropriate place for mindfulness practice	“Though I try my best [to find a place] because of the noise from family at home, it isn’t always easy, but Okay.” [Participant A]
Anticipated difficulty finding the time to practice mindfulness	“...You may need it more during study and exam periods, but maybe the time is tight, and you feel like you’re almost catching up with primary things to do in your day in relation to work,...studying,...family. You don’t have enough time.” [Participant A]
Good access to technology but varied network connectivity	“Yes, it’s available, and also easy.” [Participant C] “I just have reservations about the technical issues aspect...any online course I take or any conferences I attend, I encounter technical problems in this matter. So, I assume that this is an obstacle unless...no internet connection is needed.” [Participant N]
Social opportunity	
Influence of social environment on mindfulness practice	“I only know two; one from my family and a friend.” [who practice mindfulness] [Participant B] “I tried more than once to practice it but they [family members] didn’t like that I stay away from them, they thought that I was upset with them.” [Participant B]
Societal norms and perspectives	“...You do notice that some people, not all of them, may doubt its effectiveness because of their ignorance about it...” [Participant L] “...These are foundational concepts that already exist within us, and we already apply them...As Muslims, we already do it, but how we can be aware of it...” [Participant M]
Motivation	
Reflective motivation	
A range of anticipated beliefs about the benefits of mindfulness	“I can see its importance in reducing symptoms, whether it’s anxiety or even depression...” [Participant M] “I think...acceptance that I accept anything in my life, whether it’s pain or negative emotions...” [Participant N]
A range of experienced beliefs about the benefits of mindfulness	“...I’ve a little bit let’s say my anxiety gets higher with exams, with the due date,...but always when I feel that I started to have stress...I say and think that it’s okay, don’t think about tomorrow, you live the moment, do whatever you can do...So when I place myself in the set of the present I become better...” [Participant D]
Beliefs about the perceived side effects of mindfulness	“...In some cases, they start having anxiety like struggle to stop their thoughts, so their stress increases. Because it [mindfulness] needs time and some require immediate results...” [Participant D] “...Initially,...I tried to practice it...but there were times when I got bored and felt annoyed, and within just 2‐3 minutes, that’s it...I didn’t want to continue with the exercise anymore...” [Participant J]
Automatic motivation	
A range of emotions associated with mindfulness practice	“...I’m afraid...I don’t like doing something and not completing it or not being in the way that I want...” [Participant A]

a All themes, subthemes, and representative quotations are provided in Multimedia Appendix 2.

#### Capability

Students generally reported no health issues hindering their ability to practice mindfulness or engage in online platforms, except for minor difficulties such as physical injuries or low vision, which they could adjust for. One notable aspect within psychological capability was the variation in *knowledge of mindfulness* among students. There was a varied understanding of the concept of mindfulness, ranging from familiarity to contradictory views of its principles, or not knowing about mindfulness. Students also had a varying understanding of who mindfulness is suitable for, with references made to everyone, those with stress, or people facing mental or physical difficulties.

Another reported barrier to engaging in mindfulness practice was the variation in students’ ability to concentrate and attend for specific periods of time. Half of the students (7/14) found this challenging, whether they had experienced these difficulties during mindfulness exercises, anticipated such challenges, or noticed them during other activities (eg, while watching shows). They mentioned several reasons for this, such as their mind wandering. However, 3 students with previous experience in mindfulness observed that both concentration and attention could improve with consistent practice. Conversely, most students (11/14) demonstrated good time management skills; for example, they shared various strategies for their usual way of organizing their schedules and potential plans to allocate specific times and days for mindfulness.

#### Opportunity

As part of the opportunity afforded by the *physical environment*, students emphasized that finding or expecting to find a suitable place for mindfulness practice was generally manageable. However, they also mentioned the challenges of accessing such a place due to the presence of others in the home. Finding time to allocate specifically for mindfulness practice was more commonly anticipated to be challenging, given the tightness of students’ university schedules alongside other personal commitments. Students also stressed potential distractions from their surroundings that might negatively impact their mindfulness practice, such as social media and smart devices. Turning to the aspect of *technology*, while all students acknowledged having access to the appropriate equipment to engage in online platforms, 3 reported challenges with network connectivity, which may affect the quality of the materials received.

Regarding the *social environment* that influenced mindfulness practice, students acknowledged that mindfulness practice was uncommon among their family and friends, highlighting that they knew few people (eg, none or only 2 people) in their immediate circles who actively engaged in mindfulness. However, the extent to which others’ opinions about mindfulness might affect students’ own practice varied. Most students (11/14) considered mindfulness practice a personal choice, and had they experienced its benefits, they would have become less concerned about others’ opinions. Two students mentioned that if they were to have encountered criticism from people around them, they would have preferred to practice it privately without their knowledge to avoid such issues. Students also either experienced or anticipated receiving different levels of support from family and friends regarding their mindfulness practice. For example, 3 out of 14 students noticed that their family members were less supportive of their mindfulness practice due to reasons such as not understanding what the students were doing or being slightly annoyed by the students’ spending time alone. On the other hand, 4 students found that their families were not only supportive but also eager to join in.

Beyond their immediate social circle, *societal norms and perspectives* also influenced students’ engagement. Many students (10/14) acknowledged a general lack of awareness about mindfulness within society, indicating there was no clear understanding of what mindfulness is and its potential utility, which may impede its acceptance (eg, associating mindfulness with triviality, which might lead to a skeptical attitude toward mindfulness). They also emphasized the need for greater societal awareness of mindfulness. Three students admitted that they, too, were initially skeptical due to not knowing what mindfulness was until they gained a deeper understanding. However, 4 students mentioned that society nowadays, particularly the younger generations, is more aware of mindfulness and is more accepting of it. Another substantial group of students (12/14) acknowledged that mindfulness aligned with either Arabic culture, Islamic values, or both. They emphasized that many of its principles, once prompted to think about them, reflected what they and people in society were already doing and had been taught to do, such as practicing kindness and appreciation.

#### Motivation

Students held diverse perceptions of *anticipated benefits of mindfulness*, ranging from reductions in psychological distress (eg, stress), improvements in well-being, flexibility, acceptance and appreciation, and self-awareness, to broader expected benefits such as improvements in concentration and attention. Some of these were also reflected in students’ *experienced benefits of mindfulness*. They reported that it helped in managing stress, anxiety, and physical pain and felt more aware and connected to the present moment. Conversely, 4 students pointed out the *perceived negative side effects of mindfulness*. Three of them highlighted that mindfulness may not be suitable for those seeking immediate results, people who are self-conscious about their bodies, or those who have panic attacks. Two of these students also found the practice of mindfulness annoying and boring.

Turning to students’ *intentions to engage in mindfulness practice*, participants anticipated the importance and need for mindfulness in various situations, emphasizing its relevance, for example, when feeling overwhelmed, in daily routines, and during moments of enjoyment. Their intentions to engage in online MBIs, however, varied. Of the 14 students, 9 were eager to join such programs, recognizing them as a valuable skill to learn and build upon. In contrast, 4 students felt they had already acquired the necessary skills, making participation less important, while 1 student preferred face-to-face interactions for learning mindfulness.

Furthermore, students mentioned several *perceived facilitators of mindfulness practice* that could enhance their motivation to engage in mindfulness or maintain their practice. Examples included practicing with others to create a shared experience and aligning mindfulness practices with cultural contexts. Two students highlighted how anchor points, such as starting with breathing or focusing on certain bodily sensations, helped them or could help others stay present during mindfulness exercises. Shifting the focus to automatic motivation, 3 key *emotions associated with mindfulness practice* were identified among students: anticipated fear of failure and not completing practices properly, excitement about engaging in mindfulness, and enjoyment experienced from previous mindfulness practice.

### The Cultural Adaptation Framework by Bernal et al

#### Overview

Cultural adaptations that may facilitate students’ engagement with MBIs, with a particular focus on online versions, were addressed via the framework by Bernal et al [22]. Table 3 shows the domains, key themes, and relevant quotations. All themes and corresponding quotations are provided in Multimedia Appendix 3.

**Table 3.** Key findings of the cultural adaptation framework by Bernal et al [22].^a^

Domains and themes	Quotes
Language	
Variation in language preferences	“Arabic is better because it’s our language; expression is better in it. As for the English language, it requires specific expressions and you struggle to find the right words, which can be somewhat challenging.” [Participant E]
People	
Varying preferences for the gender of instructors	“...It doesn’t matter to me who the instructor is; I feel that...the tone of the voice is more important than the speaker’s gender...” [Participant H]
Metaphor	
Incorporating metaphors associated with Saudi and Arab culture	“...Examples in mindfulness always seem to relate to the lives of foreigners...you can’t really use these examples into the lives of Saudis. Even the example of the train—we don’t have a train. Yes, I like the example, but it’s weird for me to tell someone [about it]. Well, I don’t have a train in my life anyway to watch, how you want me to imagine it...I feel it’s nice to translate examples into things that happen in the real lives of Saudis or Arabs in general.” [Participant N]
Varied viewpoints in using Islamic-related metaphors	“Maybe in our religion. We can find in the Sunnah an-Nabawiyyah [the actions, sayings of Prophet Muhammad (peace be upon him)]...” [Participant O]
Content	
Acceptability of mindfulness exercises	“I feel this concept [appreciation] is very beautiful and very good, and I don’t feel that it needs any adjustments—like it’s already good as a whole. And, I even feel that it fits with our culture and environment, and the age you’re talking about for people at the university.” [Participant D]
Goals	
Variation in students’ goals for the online MBIs^b^	“Yes, stress...and how they deal with the negative and adverse effects. When they encounter a distressing situation, how do they bring mindfulness to that moment and manage to cope with the situation.” [Participant O]
Concept	
Students’ perspective on mindfulness aligned with its core principles	“...I might explain to them [students]...that it’s about you focusing on your awareness from all aspects—your emotions, sensations...whether internal or external, in the present moment without any interruptions from life...and it doesn’t mean you need to make any interpretations of the sensations you are feeling or seeing.” [Participant D]
Method	
Variation in the preferred online platform	“I feel an application, if there is an application specifically made for it, it will be much better...” [Participant A]
Preferences for receiving reminders and prompts	“...I feel it’s really important because...It’s nice...to have something that reminds you to bring your attention and focus.” [Participant J]
Varying preference about the duration of the online MBI and mindfulness exercises	“I prefer to have both options honestly. I’d like beginners to have a short duration to try it out, and specialised individuals want more...” [Participant N]
Context	
Factors facilitating mindfulness implementation within Saudi society	“That it focuses in our believes. Beliefs of Saudi, that would be more suitable.” [Participant O]
Factors facilitating mindfulness implementation within university setting	“...Maybe have a bonus or incentive from teachers to encourage attendance for this course initially. Not everyone might be interested at first, but I think if there’s a motivator, it can help students consider taking this course.” [Participant N]

a All themes and representative quotations are provided in Multimedia Appendix 3.

b MBI: mindfulness-based intervention.

#### Language

Students expressed varied preferences about the language for receiving online MBIs. Many students (8/14) stressed a preference for Arabic, their native language, as it facilitates self-expression and understanding. Conversely, 5 students emphasized that English could be more suitable, particularly for terms such as “mindfulness,” which were challenging to translate and comprehend in Arabic. Three students also expressed interest in receiving materials in both languages, indicating a potential need for flexibility.

#### Person

Most students (11/14) expressed that the gender of the person delivering the mindfulness audio recordings was not significant to them. They prioritized factors such as the quality and clarity of the voice and tone. However, 2 students mentioned a preference for a female voice. Three students also expressed a preference for written Arabic to be personally addressed to them rather than presented in a general manner.

#### Metaphor

A notable observation among students was the positive attitude toward using metaphors associated with Saudi and Arab cultures. Students emphasized that the closer the illustration aligned with the culture of the participant, the better. However, views varied with respect to the appropriateness of incorporating Islamic-related metaphors into an online MBI.

#### Content

At the start and during the interviews, students were given examples of mindfulness exercises (eg, sitting meditation and body scan) to introduce them to different concepts within mindfulness. Students acknowledged the acceptability of various mindfulness exercises (eg, body scan and loving kindness), emphasizing the suitability and likability of these exercises. Almost all students (13/14) also highlighted the appropriateness and acceptability of these exercises within the cultural context or university environment. However, whether based on their previous experiences or when presented with these exercises, 4 students emphasized the importance of improving awareness about mindfulness concepts and exercises, as it facilitates students’ acceptance, particularly when the exercises may be unfamiliar to them.

#### Goals

The potential goals and reasons why students undertook, or were willing to undertake, mindfulness practice varied considerably. Examples included improving their understanding of what mindfulness is, improving psychological well-being, managing psychological distress (eg, coping with stress), being more aware and connected to the present moment, making mindfulness a daily habit, and improving academic performance (eg, concentration).

#### Concept

Generally, students defined mindfulness in similar terms to its typical formal definition, including aspects such as paying attention to the present moment without judgment. However, students stressed the need for clarity in the meaning of mindfulness and identified key principles that can be used to clearly illustrate it. Examples included providing a precise definition with real-life examples, simplifying the meaning of nonjudgment, addressing misconceptions (eg, mindfulness not being merely relaxation), and normalizing potential challenges in the initial stages of mindfulness practice (eg, mind wandering).

#### Method

Students showed diverse preferences for the online platform through which they receive the intervention. Of the 14 students, 7 favored software applications, while 4 leaned toward external platforms, such as a website or a university’s online learning platform. Opinions on using the university’s platform, however, were mixed: 3 students viewed it as convenient for integrating the intervention alongside academic resources, whereas 1 felt that the intervention better remains separate to allow disengagement from study. Students also highlighted the appropriateness of receiving reminders and prompts to practice mindfulness when participating in online MBIs. However, 3 students pointed out that overly frequent reminders might not be ideal. Furthermore, students expressed varied intentions to engage in online peer-support forums.

When it came to the duration of the mindfulness course, students demonstrated a range of preferences. Of the 14 students, 6 favored brief courses (≤4 weeks), while 4 preferred longer ones (>4 weeks). One student stressed the importance of flexibility, suggesting the inclusion of both options. These preferences also extended to the mindfulness exercises, where students indicated a preference for either brief or longer sessions or having the flexibility to choose between the two options.

#### Context

Students highlighted various factors facilitating the implementation of mindfulness and online MBIs within the context of Saudi society. They emphasized the importance of integrating mindfulness into the lives of Saudis by aligning it with social habits and values, having it presented by Saudi individuals, and increasing awareness in society (eg, via social media). Students also highlighted facilitating factors within a university setting. They emphasized the crucial role of spreading awareness about mindfulness through social media and effective advertising within the university itself to reach a large proportion of students. Students also stressed the significance of university involvement in encouraging mindfulness practice. Two students also discussed how aligning this intervention with mental health could either facilitate or hinder students’ engagement in mindfulness.

### Implications for Mindfulness-Based Interventions

A series of recommendations for developing an MBI, with a specific focus on an online version, for Saudi university students are outlined in MultimediaAppendices 4  5. These recommendations aligned with the findings identified from the COM-B and the cultural adaptation framework by Bernal et al [22].

Broadly, recommendations based on the COM-B, TDF, and the Theory and Techniques Tool included specific directions that may facilitate students’ engagement with MBIs, further detailed in Multimedia Appendix 4. These included but were not limited to providing sufficient information and guidance (eg, on mindfulness practices and how to create a suitable environment for their practice), providing students with strategies and skills to conduct their own mindfulness practice more effectively and overcome obstacles (eg, finding time, improving concentration, and incorporating mindfulness into their daily routines), and providing students with resources to participate effectively in the intervention (eg, how to seek social support from people around them).

Similarly, recommendations based on the cultural adaptation framework by Bernal et al [22] consisted of practical steps to accommodate the preferences, needs, and cultural viewpoints of Saudi university students in the development of the MBI, as detailed in Multimedia Appendix 5. These included, but were not limited to, identifying essential mindfulness components and exercises that need to be incorporated into the intervention to align with students’ goals (eg, incorporating compassion and kindness), adapting mindfulness-related elements that resonate with students’ cultural backgrounds (eg, using Arabic language and using metaphors and examples relevant to the local culture), incorporating specific practical procedures for intervention delivery (eg, considering the gender of the instructor and allowing flexibility in exercise durations), tailoring the way in which the concept of mindfulness is presented to students (eg, clarifying the concept of “nonjudgment”), and supporting the integration of mindfulness practices into Saudi society and university settings (eg, using social media to raise awareness about mindfulness).

## Discussion

### Interpretation of Findings

This study adopted a qualitative exploratory design to gain in-depth insights into Saudi female university students’ perspectives on MBIs, enabling the identification of potential facilitators, barriers, and cultural factors that could enhance students’ engagement with MBIs during this formative phase. Qualitative approaches have previously been used to inform the development of MBIs for university students [5,6], particularly when little is known about how such interventions are implemented [33], as was the case in this study. This study applied the COM-B framework [7] and the cultural adaptation framework by Bernal et al [18,22], adopting a theoretically informative lens to guide data interpretation and to develop culturally sensitive recommendations for MBI design aimed at enhancing students’ acceptability of and engagement with the intervention. Overall, Saudi female university students were positively inclined toward MBIs, both specifically as students and within their wider cultural context. Their responses reflected numerous themes drawn from both frameworks, which may either facilitate or hinder engagement with the intervention and potentially improve its cultural suitability. These findings are recommended to be integrated into MBI development to accommodate the needs of Saudi female university students.

Knowledge and awareness about mindfulness were identified as a key theme in both frameworks. Students highlighted the need for greater awareness of mindfulness across various contexts, including their own understanding of mindfulness and its benefits, as well as broader societal awareness of the concept. Similar findings have been reported in previous studies, where health care staff described how increased awareness of the effectiveness of mindfulness could enhance their engagement with a self-guided MBI [34]. Furthermore, enhancing general awareness of mental health in Saudi society has been stressed as crucial for improving the uptake of psychotherapies, including MBIs, and perhaps further reducing potential barriers to engagement, such as minimizing stigma [19]. Raising mental health awareness in Saudi society may not only facilitate engagement with psychotherapies but could also break down other barriers to seeking help. Social obstacles such as stigma, cultural and family attitudes, negative perceptions about mental health difficulties, and a lack of awareness about accessing mental health information and services were identified as perceived hindrances to seeking mental health support among adults in KSA [35-40]. Promoting mindfulness awareness within the targeted population and broader Saudi society might facilitate acceptance and engagement in mental health care in KSA. Further research is needed to explore this.

Furthermore, a significant perceived obstacle to student engagement in MBIs found in this study was time. Although students emphasized their time management skills, which may indicate their capability to effectively plan their time to incorporate mindfulness practice into their routines, they perceived that allocating specific time solely for mindfulness was still challenging. This is a common difficulty reported in many previous studies of engagement with MBIs. For example, a narrative review focusing on students’ perceptions of MBIs highlighted that university students perceived a struggle to find time for meditation outside of scheduled sessions [41]. Difficulty in finding time may also contribute to the high attrition rate of MBIs among university students. Factors that seem related to time management issues, such as scheduling conflicts, being too busy, and the demands of practice commitments, were reported by students as reasons for dropping out of MBI trials [42,43]. This challenge extends beyond student populations; for example, people with chronic conditions also described being too busy or having other commitments as barriers to MBI engagement [44]. A briefer version of MBIs (eg, 100 minutes per week) has been suggested to address time commitment issues and has been shown to be effective across different populations (eg, university students) and outcomes (eg, anxiety and depression) [45]. However, it is noteworthy that students in this study showed a mixed preference for both shorter and longer durations of MBIs, whether in terms of the number of weeks or the length of mindfulness exercises. Therefore, it might be beneficial to provide students with flexibility by offering both options, which could maximize engagement and accommodate all potential needs. In addition to this, several strategies were recommended in this study to address time-related challenges when developing MBIs. Some of these recommendations align with suggestions from others, such as incorporating mindfulness practices into students’ everyday tasks [41]. Future research could further explore and assess the adoption of these strategies.

The findings of this study indicate that it might be important to align MBIs with the cultural background of the target population. This may include adapting the content to be more culturally relevant, for example, by integrating local values, metaphors, and language into the interventions. Generally, the need for cultural adaptations in designing MBIs for university students has been emphasized [41]. This need was further emphasized by how students perceived nonadapted MBIs. For example, university students in the United Arab Emirates, a Gulf Cooperation Council country that shares cultural, linguistic, and regulatory similarities with KSA, reported that incorporating metaphors related to their culture and receiving the intervention in Arabic would have helped them better connect with an MBI and enrich their mindfulness experiences [46]. Furthermore, a qualitative study conducted in Indonesia, which differs culturally from KSA but shares Islam as the majority religion, explored university students’ and relevant stakeholders’ perspectives on developing an online MBI highlighted the need to culturally adapt intervention content (eg, by incorporating Indonesian social activities and translating materials into Indonesian) [47]. The study subsequently found that an adapted version of MBI was acceptable to Indonesian students, as evidenced by a 70% completion rate. There were also preliminary improvements in students’ levels of mindfulness, distress, and well-being at postintervention [48]. Similarly, outside the student population, African women highlighted the importance of incorporating African cultural values and using culturally familiar language to enrich an MBI after receiving a nonculturally adapted version [49]. These findings from diverse contexts highlight that sociocultural factors may play a key role in shaping the acceptability and engagement of MBIs. However, it is noteworthy that, to the authors’ knowledge, no studies have directly compared the feasibility, acceptability, engagement, and effectiveness of culturally adapted MBIs to nonadapted versions. Therefore, the extent of the usefulness of such adaptations remains uncertain. Future studies should explore the incorporation of cultural adaptations into MBIs, as well as the acceptability and effectiveness of culturally adapted MBIs compared to nonadapted ones with respect to student engagement and outcomes improvement.

Furthermore, it is important to consider participant characteristics, as these may have shaped the findings and, consequently, influenced how the intervention recommendations were developed. For example, all participants were female and self-identified as Saudi nationals, which may have informed their perspectives on MBIs and, in turn, influenced the study findings. Nonetheless, given that Saudi nationals constitute more than half of the total population (57%) [50], and that the education system in KSA is typically gender-segregated, these characteristics remain relevant to the context of Saudi universities. However, further research is needed to include a more diverse cohort of students in KSA (eg, male participants and non-Saudi nationals) to enhance the transferability of findings.

To the authors’ knowledge, it remains unclear whether the evidence-based theoretical frameworks used in this study (eg, the COM-B model, TDF, and the cultural adaptation framework by Bernal et al [22]), most of which were originally developed in Western contexts, are fully applicable to the Saudi cultural setting. As this study is the first to apply theory-driven approaches to exploring MBIs among Saudi university students, it provided an opportunity to reflect on how these frameworks align with local sociocultural contexts. For example, it has been emphasized that the COM-B model does not explicitly account for the cultural background of participants (eg, their beliefs or gender), which may be relevant when designing BCTs [51]. This, in turn, may introduce subjectivity and inconsistency in determining whether and how cultural factors influence specific behaviors when designing interventions [51]. Consequently, there is a need to further test, develop, or adapt theoretical frameworks that are specific to the Saudi or broader Arab context and informed by experts familiar with regional populations and mental health systems. Whether such context-specific frameworks would prove more appropriate than those developed in Western contexts remains uncertain and represents an important direction for future research.

Another area of consideration relates to the fact that some recommendations made in this study are already incorporated into many MBIs (eg, addressing common misconceptions and normalizing experiences and emotions). Since this study followed a systematic approach to identify factors that may influence behavioral change, it suggests the importance of including these elements in MBIs for university students. This study, however, identified several other strategies based on the inclusive selection method of relevant BCTs, which aligned with anticipated barriers and facilitators that may facilitate the uptake of MBIs for students (eg, guidance on how to seek support from family and friends). Consequently, these strategies need to be tested. Future research should consider these implications, which could impact MBI engagement among Saudi university students, and then assess their feasibility, acceptability, and effectiveness.

From a wider perspective, it has been emphasized that the cultural adaptation of evidence-based interventions can be strengthened by embedding them within implementation science frameworks [52]. Such frameworks provide a contextual lens for evaluating institutional readiness, policy alignment, and organizational capacity factors crucial for ensuring that interventions are not only culturally appropriate but also realistically deliverable within existing systems [52]. In Saudi universities, institutional readiness may play a key role in the integration of culturally adapted MBIs. This includes the willingness of institutions to embed such interventions within existing student well-being systems, promote them through official university channels, and raise awareness of mindfulness as a mental health approach among students and staff members. Future studies should therefore adopt a broader implementation plan that directly engages university-level stakeholders when introducing MBIs in KSA. Examples include identifying relevant policy strategies, such as developing institutional guidelines and establishing regulations to support program delivery and promotion [12].

Furthermore, given the high demand for mental health services typically observed in university settings [53,54] and the limited availability of MBI research in KSA, universities in KSA should consider initiating pilot programs to explore the feasibility, acceptability, and preliminary efficacy of culturally sensitive mental health interventions to support student well-being. Self-guided MBIs, particularly online versions, can be well suited to higher education settings, where large numbers of users can access them at low cost [28]. This approach aligns with the broader objectives of Saudi Vision 2030, which emphasizes improving access to tailored mental health services as part of national development efforts to enhance health care [55].

### Strengths and Limitations

To the authors’ knowledge, this is the first study to explore factors influencing engagement in MBIs and their cultural appropriateness for Saudi university students. One strength of the study lies in the methodology used, which was based on well-established frameworks, guiding the development of interview topics, data analysis, and MBI recommendations. However, this study has several limitations. Participants were all female and recruited from a single women-only university due to resource limitations. This increased the likelihood of cultural homogeneity among participants, restricting the perspectives to solely female Saudi students in one location. Consequently, valuable insights from male counterparts or students in other parts of KSA need further exploration.

Additionally, the original dataset in Arabic was analyzed and translated by only one researcher, and only 20% of the English-coded quotations were independently reviewed by two reviewers. Despite these measures, some relevant data may have been missed or misunderstood during translation or coding, which could affect the study’s conclusions. Useful insights might have also been gained if the study had engaged students throughout the research process, such as involving them in developing the interview topic guides, identifying final themes, and drawing recommendations. This approach could have enhanced the relevance of the recommendations identified in the study.

Furthermore, while the mixed inductive-deductive thematic analysis used in this study aligned with its aims, the back-and-forth transition between deductive and inductive analysis could have introduced research biases (eg, overfitting within the theoretical frameworks), which could affect the study’s findings. Despite the importance of considering prior mindfulness experience (since the adoption of MBIs may vary depending on the level of prior experience), comparisons between participants with and without prior experience were deemed inappropriate for this study. Although more than half of the participants in this study self-reported prior mindfulness experience, this varied from completing MBI courses and studying mindfulness as part of their education to engaging with it solely through reading booklets or watching online videos. This diversity meant that only a small number of participants (5/14, 36%) demonstrated sufficient familiarity with mindfulness to allow meaningful comparison between those with and without formal mindfulness experience. Future research should examine the impact of participants’ prior experiences with mindfulness on the barriers to and facilitators of engagement with MBIs. Lastly, given the specific focus of this study on online MBIs, some influential factors that may be applicable to face-to-face MBIs engagement discussed in previous studies (eg, the role of facilitators and online vs face-to-face delivery) were not addressed. Future research should explore these factors to enhance the design of MBIs to meet students’ needs more effectively.

### Future Research

This study provides potentially useful insights for developers of MBIs targeting KSA university students. This study is part of a larger project that follows the Medical Research Council framework for developing and evaluating complex interventions, which involves synthesizing evidence to inform intervention design [56]. Building on the findings of this study, the next phase will involve a mixed methods, uncontrolled feasibility study to assess the acceptability, feasibility, and preliminary signals of efficacy of a culturally adapted online MBI designed for Saudi female university students. If the intervention proves feasible and acceptable, a subsequent RCT with an internal pilot phase could be conducted to evaluate its effectiveness on a larger scale.

Furthermore, given the wide range of recommendations generated in this study, it may be neither feasible nor advisable to implement all identified strategies in future applications. Typically, behavioral interventions adopt a systematic process to prioritize and select the most appropriate BCTs and intervention strategies when designing the intervention [12]. One such approach involves applying the APEASE (acceptability, practicability, cost-effectiveness, affordability, safety, and equity) criteria [12]. These criteria can be systematically applied and evaluated in collaboration with relevant stakeholders (eg, psychologists and university staff) to guide the refinement of the culturally adapted MBI, which can then be tested in future research.

### Conclusions

This study explored factors that could facilitate or hinder Saudi female university students’ engagement with MBIs and their perspectives on culturally appropriate adjustments, using theory-informed approaches. Subsequently, systematic recommendations were formulated to integrate into the development of an online MBI. These recommendations aim to enhance the feasibility, acceptability, engagement with, and effectiveness of MBIs among Saudi university students, particularly female students. However, whether they do in fact achieve these aims is unknown. Future research should seek to evaluate this further.

## Supplementary material

10.2196/78532 Multimedia Appendix 1 Interview topics based on the COM-B framework and the cultural adaptation framework by Bernal et al. COM-B: Capability, Opportunity, and Motivation Domains of Behavior Change.

10.2196/78532 Multimedia Appendix 2 Findings of the COM-B framework with further illustrative quotations. COM-B: Capability, Opportunity, and Motivation Domains of Behavior Change.

10.2196/78532 Multimedia Appendix 3 Findings of the cultural adaptation framework by Bernal et al with further illustrative quotations.

10.2196/78532 Multimedia Appendix 4 COM-B mapped recommendations using the Theory and Techniques Tool. COM-B: Capability, Opportunity, and Motivation Domains of Behavior Change.

10.2196/78532 Multimedia Appendix 5 Recommendations from the cultural adaptation framework by Bernal et al.

10.2196/78532 Checklist 1 COREQ checklist.

## Abbreviations

APEASE: acceptability, practicability, effectiveness/cost-effectiveness, affordability, side effects/safety, and equity criteria
BCT: behavior change technique
COM-B: Capability, Opportunity, and Motivation Domains of Behavior Change
COREQ: Consolidated Criteria for Reporting Qualitative Research
KSA: Kingdom of Saudi Arabia
MBI: mindfulness-based intervention
RCT: randomized controlled trial
TDF: Theoretical Domains Framework
