# Supplementary material for: Facilitators, Barriers, and Cultural Appropriateness of Mindfulness-Based Interventions Among Saudi Female University Students: Qualitative Study
Source: JMIR Form Res. 2025 Dec 19;9:e78532. doi: 10.2196/78532 (PMC12716633; doi:10.2196/78532)
Supplement: Multimedia Appendix 1 [file formative-v9-e78532-s001.pdf]

Interview topics based on the COM-B framework and the cultural adaptation framework by Bernal et al

COM-B Domain	Key questions
**Behaviour**	• What is your previous and current experience of mindfulness or meditation?<br>○ Prompt: Have you ever completed a mindfulness or meditation course or practiced mindfulness or meditation exercises? To what extent is mindfulness or meditation something that you usually practice?<br>○ If yes: Can you tell me more about that? What format was it in (prompts: self-help/formal training, group/individual setting, in-person/online)?
**Capability (physical)**	• Do you have any physical health conditions that could impact on your ability to practice mindfulness or meditation exercises?<br>• Do you have any physical health conditions that could impact on your ability to complete an online course?
**Capability (psychological)**	• What is your understanding of mindfulness or meditation?<br>• Who do you think mindfulness and meditation exercises are for? (Prompt: Are mindfulness and meditation for all people or only those in need e.g., those who have mental health issues?)<br>• Can you tell me about any MBIs/online MBIs that you are aware of at the university? (Prompts: Who can access these? When, where, and how can you access these?)<br>• Mindfulness or meditation exercises typically involve paying attention to your thoughts, feelings and sensations for a set amount of time. How easy would you find this? (Prompts: What might help? What might get in the way?)
**Opportunity (physical)**	• Interviewer describes the ideal environment for engaging in an online mindfulness or meditation: How easy would it be for you to access that type of environment? (Prompts: What might help? What might get in the way?)<br>• Interviewer describes the appropriate technology needed to use online mindfulness or meditation: How easy would it be for you to access the appropriate technology or resources to use online mindfulness or meditation? (Prompts: devices, Internet connection? What might help? What might get in the way?)<br>• How would you juggle practicing online mindfulness or meditation exercises alongside other demands on your time? (Prompts: What might help? What might get in the way?)
**Opportunity (social)**	• If you were to practice mindfulness or meditation, how do you think your friends/people around you would support or encourage you to do this?<br>• What do you think your friends/people around you would say if you practiced mindfulness or meditation, or took part in an online mindfulness or meditation course? (Prompts: How do you think they would feel about this?)<br>• How many of your friends/people around you currently practice mindfulness or meditation?<br>• To what extent would your friends/people around you influence whether or not you practiced mindfulness or meditation? How much of an effect would their opinions about mindfulness or meditation have on you?
**Motivation (reflective)**	• Why do you think people practice mindfulness or meditation?<br>• How important do you think practicing mindfulness or meditation is?<br>• Overall, how useful do you think practicing mindfulness or meditation would be for you?<br>• What downsides might there be of practicing mindfulness or meditation?<br>• In what circumstances would you practice mindfulness or meditation?<br>• How likely is it that you would practice mindfulness or meditation? If there was an online MBI that was adapted for KSA students, how likely is it that you would use this?

	• How confident would you feel about practicing mindfulness or meditation? How confident would you feel about taking part in an online course?
**Motivation (automatic)**	• How would you feel about practicing mindfulness or meditation or using an online mindfulness and meditation course? (Prompts: Would you have any worries or concerns about it?)<br>• How could you incorporate an online mindfulness or meditation course into your daily routine? How could you make it so that it became part of your daily routine? (Prompts: What might help? What might get in the way?)<br>• What kind of things might keep you motivated and encourage you to continue practicing mindfulness or meditation or using an online mindfulness or meditation course?
**Domains of the cultural adaptation framework by Bernal et al**	**Key questions**
**Language**	• If you were to practice mindfulness or meditation, in which language would you prefer it to be? Arabic or English? (Prompts: In relation to materials-e.g., audio recordings, texts, stories, sayings, pictures)<br>• What are your preferences with respect to the Arabic language?
**People**	• Thinking about who would guide mindfulness or meditation exercises, and what type of language they would use to refer to other people, what would be important to you? What kind of things would help you practice mindfulness or meditation or take part in an online MBI in relation to people? (Prompts: female/male voice, mixture of different people guiding exercises?)
**Metaphor**	• What kind of metaphors/sayings do you think we should include in an online MBI? (e.g. metaphors related to Saudi culture, Arab, Islam)<br>• Can you give examples? (Prompts: authors, books, etc.)
**Content**	• One mindfulness exercise involves XYZ. How would you feel about being asked to do this? Any thoughts or concerns? How can we adapt it to Saudi culture?<br>• Another mindfulness exercise involves XYZ. How would you feel about being asked to do this? Any thoughts or concerns? How can we adapt it to Saudi culture?<br>• Etc
**Goals**	• What do you hope an online MBI could be used for? (Prompts: Goals can be related to academic-related stress, psychological wellbeing, etc.)<br>• If we were to develop an online MBI for KSA university students, what do you think its main aims should be?
**Concept**	• In your own words, how would you describe mindfulness or meditation to KSA university students?<br>• What would it be important to say or not say when describing mindfulness or meditation to KSA students?
**Method**	• If we were to design an online MBI for KSA students, what do you think this should look like?<br>• Which online platform would you prefer and why? (e.g. software application, University blackboard, website)<br>• What equipment, if any, would help you to take part in an online MBI?<br>• How would you feel about being given reminders to complete daily or weekly mindfulness exercises? (Prompts: The purpose of reminder, delivery, frequency)<br>• How would you feel about having an interactive forum within an online MBI? Would you take part in it? Why/why not?<br>• What length would you prefer an online MBI to be? (e.g., 10 days, 2 weeks).<br>• What length would you prefer mindfulness exercises to be? (Prompts: Shorter exercise [3-5 minutes], longer [10-15 minutes])
**Context**	• From your own experiences and/or thoughts, how could an online MBI be integrated into Saudi culture?<br>• From your own experiences and/or thoughts, how could an online MBI be integrated into university life?

Note: MBI= mindfulness-based intervention; KSA= Kingdom of Saudi Arabia; references for frameworks a) Bernal, G., & Sáez-Santiago, E. (2006). Culturally centered psychosocial interventions. *Journal of Community Psychology*, 34(2), 121–132, and b) Michie, S., van Stralen, M. M., & West, R. (2011). The behaviour change wheel: A new method for characterising and designing behaviour change interventions. *Implementation Science*, 6(1), 42.
