# Supplementary material for: Facilitators, Barriers, and Cultural Appropriateness of Mindfulness-Based Interventions Among Saudi Female University Students: Qualitative Study
Source: JMIR Form Res. 2025 Dec 19;9:e78532. doi: 10.2196/78532 (PMC12716633; doi:10.2196/78532)
Supplement: Multimedia Appendix 2 [file formative-v9-e78532-s002.pdf]

Findings of the COM-B framework with further illustrative quotations

Physical capability	
**Statement**	**Example Quotes**
No significant health issues impacting mindfulness practice or online course use.	I don't have *AlhumdAllah* [thank God] (A)
	I have a low vision, but I wear glasses. (G)

Psychological capability		
**Theme**	**Subtheme**	**Example Quotes**
Knowledge of mindfulness	Variability in the understanding of what mindfulness is.	Honestly, I've never heard about mindfulness before, I don't think I've tried it. (A)
		Maybe it's the person' ability to be present in the moment without being influenced by negative judgments or experiencing negative emotions towards them. They can be at ease in the moment, focused on it, and not dwell on the past. (O)
		Maybe it makes you getting busy from the reality, living more inside, in your thoughts, I don't know maybe this is something negative. (A)
	Varied understanding of who mindfulness is appropriate for.	…there may be specific groups of people who, for example, have high anxiety, high levels of worry, or those who have obsessions. (J)
		… I've read a lot about the topic of chronic pain. People who have chronic health issues may experience some improvement in their quality of life since they may not be able to eliminate this problem. So at least it could improve their quality of life. (N)
		I feel it's for busy people such as students, employees. (B)
		All people. (G)
	(lack of) Knowledge about mindfulness courses offered by university.	I don't know unfortunately. (A)
		I remember that I saw a course but I don't remember the doctor or the details generally. (C)
		I don't think it is easy, especially for people who easily get distracted… (A)

Cognitive skills needed to engage in mindfulness practice.	Varied ability to attend and concentrate..	…I've difficulty sustaining prolonged attention or maintaining focus for a long time. There are many things of course that could impact "what occupies person's mind" " what the one's thinking of" and "what's on one's mind". No matter how hard I try, for example, to avoid thinking about anything other than the place that I'm in or what I'm hearing, suddenly something happen like scatter. I find it extremely difficult to maintain my concentration on nothing I've to occupy my mind with something… (K)
		For me, it is easy. But sometimes it depends on the time, whether there are things, it depends on the day, for example. But generally, I can, and able to pay attention. (H)
		Honestly, in the beginning, it was really difficult for me to stay focused for five minutes on the present moment. But with practice, certainty it does make a difference. Mindfulness, for the most part, is a practice. That we train ourselves so our nervous system get trained and becomes more attentive to the moment… (M)
	Good time management	It's fine, I can divide the time and allocate some for it, just like when I allocate time for studying, prayer, and self-care. I can incorporate it into my schedule. (O)
		I've a morning period, so I wake up a few hours before work to dedicate that time to reading, walking, skiing, or any activity I want to do. If I want to add anything usually, I do it during this period. (H)
		I'm saying that considering our current university situation, we could allocate a day during the weekend for it ... (K)

**Physical opportunity**		
**Theme**	**Subtheme**	**Example Quotes**
Environment	Variation in access to appropriate place for mindfulness practice.	Yes, it's definitely available;…there's no obstacles. (C)
		Though I try my best [to find a place] because of the noise from family at home, it isn't always easy, but Okay. (A)
	Anticipated difficulty finding the time to practise mindfulness	Yes, of course you may need it more during study and exam periods but maybe the time is tight, and you feel like you're almost catching up with primary things to do in your day in relation to work in relation to studying in relation to family, you don't have enough time. (A)
	Distractions impacting on mindfulness practice.	For things that can make it difficult … distractions, being in a place with many distractions or having the mobile nearby. (O)

		For a year now, I've deleted social media, and I know that it affects our attention span. I believe that it someway impacts our practice of mindfulness…but I feel that this is what has helped me focus more. (H)
Technology	Good access to necessary equipment	Yes, it's available, and also easy. (C)
	Varied experiences with network connectivity	Certainly, the internet and everything else is available to me. There are no difficulties. (G)
		I just have reservations about the technical issues aspect, like right now, you see how the meeting frequently disconnects. Any online course I take or any conferences I attend, I encounter technical problems in this matter. So, I assume that this is an obstacle unless the course is pre-recorded and they send me the recorded lecture, so no need for internet connection. (N)

**Social opportunity**		
**Theme**	**Subtheme**	**Example Quotes**
Influence of social environment on mindfulness practice.	Lack of mindfulness practice among family and friends	I only know two; one from my family and a friend. (B)
		Honestly, I don't know anyone. (A)
	Impact of other's opinion on student's practice.	… I feel it is [mindfulness], how would I explain that, I feel it's a very personal, one of the deepest things that the person practice with oneself, and it is difficult that I talk and say "I practice mindfulness". (N)
		I don't think so. In other words, if I try something and feel that it benefited me, I won't feel that it has an impact. (H)
		I don't expect to stop because of their opinions, but I may reduce it in front of them. I might choose to do it in solitude to avoid distractions or criticisms. (O)
	Variation in support from family and friends	For me no, I tried more than once to practice it but they [family members] didn't like that I stay away from them, they thought that I was upset with them. (B)
		I may have faced even some sarcasms initially, like "what are you doing?"…So they were not encouraging at the beginning especially my family and friends. (M)

	for mindfulness practice.	I don't think I shared it with anyone except for my mom, and she was supportive. She wanted to practice it. (H)
Societal norms and perspectives	(lack of) awareness of mindfulness within society.	Correct, in the beginning, you do notice that some people, not all of them, may doubt its effectiveness because of their ignorance about it- means it's not a lack of their conviction but rather of their ignorance. But, once they know the true about it they become convinced. (L)
		Sarcasm… they don't even accept it.... (O)
		That's true. I also experienced it myself. At the beginning…I couldn't accept it. I was saying, "What nonsense are they doing?" But later on, I started to understand it, and in fact, I began to like these exercises… So it requires a bit more clarification and practice so I can accept this concept. (M)
		… the younger generation might be more aware of this so I think it's much accepting of the idea, they might already practising it but they don't know its term or what it's called. (N)
		…I feel our role now as psychologists is to talk more about mindfulness because it's very important….but I don't feel like there is enough awareness among people during this period. (M)
	Consistency of mindfulness with Arabic culture and Islam values	I believe that the believes that are associated to our society "AlhamdAllah" [thank God] those with good values "how I can enjoy my relationship" my community and family are important… (O)
		'…I honestly feel that it is suitable and wonderful for us to incorporate it into our culture because we kind of do some aspects of it…' (D)
		Yes and like helping, when you help someone automatically while walking, for example, someone has dopped something,… or you saw cats in the university maybe they need water or food. They are all random acts of kindness that we are automatically doing. (A)
		We have been practicing it since childhood, so I think that upbringing has had an impact on this matter… (E)
		I see the concept as truly wonderful, especially when it's explained to university students. But, we might clarify that it's actually related to the religion. These are foundational concepts that already exist within us, and we already apply them. But practicing them with awareness, "I smiled today" but being aware that I smiled today or engaged in this kindness, of what active kindness that I've done throughout my day, but I'm aware of. As Muslims, we already do it, but how we can be aware of it…(M)

Reflective motivation		
**Theme**	**Subtheme**	**Quotes**
Anticipated beliefs about the benefits of mindfulness	Self and emotions discovery.	… there’re people who want to change something about themselves and are unable to reach their true feelings. That is why they practice mindfulness, which can lead them to the fact that they were absent from themselves that they do not know it and do not realise about themselves; Yes, explore their feelings… (C)
	Reduce psychological distress	I can see its importance in reducing symptoms, whether it's anxiety or even depression. It helps the person to have greater acceptance and compassion with self, especially with anxiety, because often when we’re preoccupied with our thoughts, there is an increase in suffering, and we start to suffer and then these symptoms start to occur whether anxiety or depression. So it greatly help in this regard. (M)
	Improve psychological wellbeing and flexibility	I also feel that it enhances a person's flexibility; I feel it's one of the skills that you also have when interacting. You having a kind of flexibility when you become connected to the present moment. When you're feeling angry or experiencing uncomfortable emotions, being aware and not passing judgment can help you on your responses. (J)
		‘… It helps us to feel the joy in life, it increases the quality of life…’(O)
	Improve responses to situations	…And it enables the person to make decisions calmly and deliberately, without hastening into their decisions… (E)
	Acceptance and appreciation	I think, in general, acceptance that I accept anything in my life, whether it's pain or negative emotions. Acceptance is the general concept of mindfulness, instead of blame. And I think one of the benefits is that it reduces self-blame, for example, it allows a person to enjoy their life… (N)
		For example there are things that we lose our feeling on them when we do have them or are frequent on a daily bases, and there are people who do not have them, but we do not feel this grace/blessing… (C)
	Improve cognitive skills.	Maybe it helps with concentration and attention. (J)
	Awareness of the body and present moment.	First, I feel that it is comforting, this is the first thing can benefit us. Psychological comfort. I feel it has a connection between the person and his body. Sometimes, one may feel disconnected from oneself…the idea of being detached from one's body, he does not feel himself as a complete person. But, I feel that it helps the person return, and reunite… (H)

		For me, I think one of the things that it does is that it makes a person stays connected to the present moment. It alleviates excessive thinking and thoughts, for example. A person can be more present in the current moment and reduce overthinking and engagement with thoughts about the future or the past… (J)
A range of experienced beliefs about the benefits of mindfulness		…I've a little bit let's say my anxiety gets higher with exams, with the due date, and with these things, but always when I feel that I started to have stress, it started to increase, or that I started to get anxious, or noticed symptoms, for example, my heartbeat is increasing or so, I stop. I say and think that it is okay, don't think about tomorrow, you live the moment, do whatever you can do… So when I place myself in the set of the present I become better… (D)
		In dealing with relationships, I've been trying to use mindfulness. Especially when trying to understand what's the happening with the person. What am I currently experiencing? Am I feeling distressed? Do I have a stomach-ache?... So, I think even in relationships can be good. (O)
		…When the period comes, the pain isn't very severe, but it's annoying and continuous. So, one of the things I used to do was to use mindfulness, where I'd focus on my sensation of this pain more than I'd complain about it. Honestly, the surprise was that it worked. (N)
		…Because I'm now completing my master's degree, I'm trying my best to connect to the moment right now, as it's a wonderful moment and a pleasant experience in my life. When it goes away like this and I'm completely occupied with future matters or not fully connected to the journey itself, I feel that it's really important in terms of performance, my engagement with my environment... (J)
Beliefs about the perceived side effects of mindfulness	Negative impact on people experiencing psychological distress	In our field, not all patients are suitable for mindfulness. In some cases, they start having anxiety like struggle to stop their thoughts, so their stress increase, because it [mindfulness] needs time and some require immediate results… (D)
		Possible negative aspects of meditation in general include the fact that it might not be suitable for a specific group of people who have panic attacks or those who experience fear when they become aware of their bodies, they become terrified… For instance, I saw a person did meditation and she was already experiencing severe panic attacks. Suddenly, she got up without completing a couple of minutes, started screaming, and said she felt her heart racing…but when we searched about it, we found that there are indeed experts who recommend not to practice meditation for those who have panic attacks. (L)
	Annoying and boring	… As for me, initially, it was challenging and the concept itself was quite difficult to grasp fully. I tried to practice it, to be honest, but there were times when I got bored and felt annoyed, and within just 2-3

		minutes, that's it, I'd feel like, I wanted to close it. I didn't want to continue with the exercise anymore… (J)
Intentions to engage in mindfulness practice	Mindfulness is needed in different situations	For me, in moments when I feel overwhelmed, with emotions around, I don't really know what's happening right now. It's quite nice for me to bring my attention. I focus on what's going on without judgment. (J)
		But maybe during times of stress in studies or when there are family issues, I feel that it can be helpful. (H)
		I need it generally on a daily basis, but most likely during days of pressure, we need it, but typically, I feel the need for it daily, on a day-to-day basis. (M)
		Mindfulness can also be used during moments of joy. It's beautiful that you enjoy the moment and what's happening right now. Having awareness, bring your attention to what's happening right now. I also feel that mindfulness can be useful not only during moments that are unpleasant. (J)
	Varying intentions to engage in online mindfulness courses..	For sure I feel high; Because I like to see what things could help me in building up things, skills within my personality. In fact, if this thing is being done for the university and academic environment, and such. I'd utilize all the things that could potentially help me. (J)
		'Yes, honestly, if the time is appropriate… not in a time of study' stress, why not I don't have any problem.' (A)
		For myself, I don't feel that, the percentage wouldn't be high, because I prefer things to be in-person. (H)
		I won't participate in it, to be honest. If it were a few years ago, I might have considered joining, but currently, I don't anticipate this [she is already practicing]. (M)
Perceived facilitators of mindfulness practice		I think that each culture is different, so if there is something specific to our culture, its benefits would be higher, but of course, after conducting studies and proving its effectiveness in our society... (D)
		The results, when I see the results, when I see how it reflected on me, that's what gives me motivation. (H)
		Yes, when the experience is shared, like shared experience it would be much easy to practice and more fun, it becomes much easier. When you are alone it would be difficult that you keep practicing it unless you have a specific commitment about mindfulness… (O)
		…The things that have helped me, honestly, to be more present in the practice itself, is starting meditation with the breath. Because the breath helps to calm the mind, so the process of meditation becomes much easier afterward. This is what I've observed from my own experience. (M)

Automatic motivation	
**Theme**	**Quotes**
A range of emotions associated with mindfulness practice	…but maybe I'm afraid that I cannot, even if the place is quiet maybe someone interrupts me, and someone distracts me, so I cannot focus completely. I don't like doing something and not completing it or not being in the way that I want, so maybe I'll take a while to find a suitable time for me. (A)
	Excited, because it's a new thing and would like to try it and I think it'll be a nice addition to me. (C)
	'…When I logged into my email and saw mindfulness, I thought "Why not give it a try?" It really excited me that it increases focus on a thing …' (F)
	… I love the affirmation. So, as soon as I wake up in the morning, I've to say affirmations to myself. After I finish the affirmations, I take 5 to 10 minutes to be present in the moment… (D)
