# Supplementary material for: Facilitators, Barriers, and Cultural Appropriateness of Mindfulness-Based Interventions Among Saudi Female University Students: Qualitative Study
Source: JMIR Form Res. 2025 Dec 19;9:e78532. doi: 10.2196/78532 (PMC12716633; doi:10.2196/78532)
Supplement: Multimedia Appendix 3 [file formative-v9-e78532-s003.pdf]

Findings of the cultural adaptation framework by Bernal et al with further illustrative quotations

Language	
**Theme**	**Quotes**
Variation in language preferences	Arabic is better because it's our language; expression is better in it. As for the English language, it requires specific expression and you struggle to find the right words, which can be somewhat challenging. But when our native language is used, it's better … (E)
	Yes, I feel that the Arabic translation might not fully convey or describe the English word... Nevertheless, I find that the English language somehow resonates with me more. (M)
	If it's for personal use, I feel that English might be better, but I also hope to become proficient in Arabic…(K)

People	
**Theme**	**Quotes**
Varying preferences for the gender of instructors	I feel like it doesn't matter to me who the instructor is; I feel that the tone of the voice is important, the tone of the voice is more important than the speaker's gender, whether it is calm or soothing to the ears (H)
	For me it is better if she was a female who deliver [the speech] (B)
Preference of personalised written language	When directed towards me, it could become deeper. (K)

Metaphor	
**Theme**	**Quotes**
Incorporating metaphors associated with Saudi and Arab culture	I feel that the closer it is, the better it is. The closer it is to person, targeted group, the better it is. (H)
	…However, for example, examples in mindfulness always seem to relate to the lives of foreigners [non Saudis]. I mean, you can't really use the example into the lives of Saudis. Even the example of the train, we don't have a train. Yes, I like the example, but it's weird for me to tell someone. Well, I don't have a train in my

	life anyway to watch how you want me to imagine it…I feel it's nice to translate examples into things that happen in the real lives of Saudis or Arabs in general. (N)
Varied viewpoints in using Islamic-related metaphors	Maybe in our religion. We can find in the Sunnah an-Nabawiyyah, I think, especially with the prophet (peace be upon him). (O)
	…I don't prefer to link it with Hadith or Quran. Maybe someone else might like this, but personally, I don't prefer it. (N)

**Content**	
**Theme**	**Quotes**
Acceptability of mindfulness exercises	I feel this concept [appreciation] very beautiful and very good, and I don't feel that it needs any adjustments, like it's already good as a whole. And I even feel that it fits with our culture and environment, and the age you're talking about for the people in the university. (D)
	It [body scan] makes me feel like it lets you focus more on your body parts, and even the connectedness in the present moment becomes stronger than with breathing, honestly. (J)
	I feel this is something daily for me [kindness] as a student. I face things, especially when I open the door…and in the elevator, who enters and who exits first. In fact, it happens very often and it's also quite pleasant. (F)
Awareness facilitating acceptance of mindfulness exercises	I see that this exercise [body scan] is quite suitable for university students, but it might be helpful initially to clarify that it's not about relaxation, it's a mindfulness. There's many people might confuse between relaxation and mindfulness practices… (M)
	…Honestly, what you mentioned is something new to me. But maybe if I try, it's fine. I might give it a try and see… (J)

**Goals**	
**Theme**	**Quotes**
Variation in students' goals of the online MBIs	Increase people's knowledge about mindfulness, its benefits and its concept. It is something a little bit new that I've never heard before though I like such topics… (A)
	Also, mindfulness might enhance the enjoyment in our lives. When I'm present in the moment, I'll feel the pleasure with greater happiness. I see this feeling as one of the most important goals. (M)

	It’s how to arrange my feelings and how do I enhance my response to them in order to live every moment by its moment… (C)
	Yes, stress… and how do they deal with the negative and adverse effects. when they encounter a distressing situation, how do they bring mindfulness at that moment and manage to cope with the situation. (O)
	It's how they deal with themselves [students] in an excellent way not just that they criticise themselves harshly… (C)
	I just feel like I want to be aware, maybe aware of my body, aware of the surroundings around me. I feel like this is my biggest goal. (H)
	I feel like the most important thing is not only in term of study but the entire journey, it is nice that the person live it… (J)
	Maybe I don't know if I'm exaggerating this point or not, but maybe improve the academic performance; like concentrating, for example… (N)
	Making meditation a routine in their lives [for students]. (B)

**Concept**	
**Theme**	**Quotes**
Students' perspective on mindfulness aligned with its core principles	I feel like as a definition. If I’m going to define it, is about living in the present moment in its true form, without magnifying or diminishing it, whether it's sad or happy. (N)
	…I might explain to them [students] …that it's about you focusing on your awareness from all aspects—your emotions, sensations, and everything you feel, whether internal or external, in the current moment without any interruptions from life… and it doesn't mean you need to make any interpretations of the sensations you are feeling or seeing (D)
Key principles for fostering mindfulness understanding	I feel without making judgments, this is the most important thing; … And I could explain without passing any judgments because I feel maybe saying it like this, no one understands what it means "Without making judgments"… (J)
	…. it is not relaxation… (M)
	I feel that the most important thing I need to say is that not a thing from the first time you practice it. They [students] must know the expectations, it's normal that the first time or even the second time, you might not be able to do it. It is very normal that the negative thoughts attack you very… (N)
	I feel it is important to give a clear definition, we mention its exercises, we mention its benefits, even its downsides… (A)

	…for example, we can use real-life examples from their daily experiences. This could make it easier to grasp the idea. (H)

Method	
**Theme**	**Quotes**
Variation in the preferred online platform	I feel an application, if there is an application specifically made for it , it will be much better, it will be very useful… (A)
	… I feel a website [is better] just log in by link and that's it… (B)
	…Blackboard, I don't think anyone will preferer it because blackboard is linked with studying, and we want to disconnect from studying. (B)
	If it's available on the Blackboard, that would be excellent because everything related to the student at the university is already on the Blackboard and the banner. When these practices at these two places, I feel that it's better than having something external, for example. (K)
Preferences for receiving reminders/prompts	…I feel it's really important because, just like you, you're bringing your attention and focus, It's nice, for instance, to have something that reminds you to bring your attention and focus. (J)
	But not too many; I can imagine that it would be nice to receive one notification during the day to remind me and gives me a word about mindfulness. (N)
Varying intentions to engage in an online peer-support forum	Yes sure sure, the interaction increases the enjoyment of the experience. (O)
	Maybe I join the group but wouldn't participate in it. (N)
	I feel it would distract me, I wouldn't pay attention to it when there is too many chat or interaction. (F)
Varying preference about the duration of the online MBI and mindfulness exercises	Not too long, because honestly, when it's long, one can get bored; Yes, two weeks is like a perfect duration, maybe a month, but anything more than that is a lot… (D)
	I prefer a longer duration. It's what I feel gives you lasting results. However, if I take something for two weeks or four weeks, I don't think it would have the same impact. (H)
	I prefer to have both options honestly. I'd like beginners to have a short duration to try it out, and specialised individuals want more, they are interested… (N)
	Also I see it shorter [mindfulness exercises], for me. (K)
	I feel half an hour is ideal [mindfulness exercises] (M)

	Yes, Here I'm saying it's optional. If you like this session five minutes, then choose it, and if you want it ten minutes, then choose it… (F)

Context	
**Theme**	**Quotes**
Factors facilitating mindfulness implementation within Saudi society	We can use the media influence, those that are currently trend, and identify the apps where people gather the most. Then, we can make advertisements, spreading awareness. (L)
	Maybe in the videos, if someone from our own culture speaks in the video, I feel that it would be really helpful. Like, when we often see someone from our culture wearing Abaia, headscarves, one would feel a bit more related and feel like the things she is saying will truly be beneficial for us…. (D)
	That it focuses in our believes. Beliefs of Saudi, that would be more suitable. (O)
Factors facilitating mindfulness implementation within university setting.	Also, from the perspective of university students…it's possible that in order for us to get them interested in the thing you want, there should be really good marketing involved, or you provide an advertisement in a way that really attracts them, something that is close to them, they're looking for, things that are closer to their current way. (D)
	…Maybe to have a bonus or incentive from teachers to encourage attendance for this course initially. At the beginning, not everyone might be interested, but I think if there's a motivator, it can help students consider taking this course. (N)
	We can talk about it as a therapy that's mindfulness is a therapy. For health specialists maybe you can combine the two, it will attract them since this is their speciality. Also when you incorporate it with mental health, these things attract me personally. (A)
	…I will not say that it is like a therapy, I'll say that it is…a training course..; Some people don't accept the word that, I'm very healthy person I don't need a therapy. (C)
