# Supplementary material for: Facilitators, Barriers, and Cultural Appropriateness of Mindfulness-Based Interventions Among Saudi Female University Students: Qualitative Study
Source: JMIR Form Res. 2025 Dec 19;9:e78532. doi: 10.2196/78532 (PMC12716633; doi:10.2196/78532)
Supplement: Multimedia Appendix 4 [file formative-v9-e78532-s004.pdf]

COM-B mapped recommendations using the Theory and Technique Tool.

Subdomain<br>• themes	Proposed Theoretical Constructs of the TDF	Proposed behaviour change techniques	Recommendations	Irrelevant behaviour techniques [reason]
		**Physical capability**		
No significant health issues impacting on mindfulness practice or online use.	NA	NA	NA	NA
		**Psychological capability**		
Knowledge of mindfulness<br>• Variability in the understandin g of what mindfulness is<br>• Varied understandin g of who mindfulness is appropriate for	Knowledge	4.1. Instruction on how to perform behaviour.<br>4.2. Information about antecedents<br>5.1. Information about health consequences.<br>5.3. Information about social and environmental consequences.	4.1. Provide information about what mindfulness is and the common misconceptions about it.<br>4.1. Provide information about who mindfulness is appropriate for and the different situations in which students can practice it.<br>4.1. Incorporate information about the available mindfulness courses onto the university's psychological support page to enable students to learn about and access these courses.<br>4.2. Provide information about the situations or triggers in which students can practice the different mindfulness exercises (e.g. practicing the coping	2.6. Biofeedback<br>[No biofeedback monitoring devices or measurements are typically used in MBIs].

• (lack of) Knowledge about mindfulness courses offered by university.			breathing space when feeling stressed or overwhelmed).<br>5.1. See reflective motivation.<br>5.3. See reflective motivation.	
Cognitive skills needed to engage in mindfulness practice.<br>• Varied ability to attend and concentrate.<br>• Good time management	Memory, attention & decision process<br><br>Skill	1.2. Problem solving<br>4.1 Instruction on how to perform behaviour<br>7.1. Prompts/cues<br>8.1. Behavioural practice/rehearsal<br>8.7. Graded tasks<br>11.3. Conserving mental resources	1.2. After trying different anchor points for mindfulness practices (e.g. feet, seat, hands, breath), advise students to determine which one helps them to refocus their attention on the mindfulness practice most effectively when their attention wanders.<br>4.1. Normalise and provide guidance on how to bring one's attention back to the present moment.<br>4.1. Provide guidance and examples of how to allocate time for mindfulness practice.<br>7.1. Encourage students to practice mindfulness via sending reminders (emails/notifications).<br>8.1. Encourage and prompt students to practice mindfulness daily and integrate it into their daily lives, including completing homework, as much as possible. Highlight the importance of regular practice in managing their attention wandering during mindfulness practices.<br>8.7. Encourage students to start with brief mindfulness exercises and gradually	NA

			extend the duration as they become more familiar with them.<br>11.3. Encourage students to choose and reflect on the best day of the week and time of day that works for them and when they feel alert for their mindfulness practice.	
		**Physical opportunity**		
Environment<br>• Variation in access to appropriate place for mindfulness practice.<br>• Difficulty finding the time to practise mindfulness<br>• Distraction impacting on mindfulness practice	Environmental context and resources.	3.2. Social support (practical)<br>7.1. Prompts/cues.<br>7.5. Remove aversive stimulus<br>12.1. Restructuring the physical environment.<br>12.2. Restructuring the social environment<br>12.5. Adding objects to the environment.	3.2. Provide practical recommendations to students on how they can seek support from family and friends during their mindfulness practice (e.g. asking family and friends to remind them to practice, and discussing with family about not interrupting during their practice).<br>7.1. Provide prompts how to find the time to practice and how to reduce the influence of distractions on mindfulness practice.<br>7.1. Bring difficulties in finding the time to practice and being distracted by social media into instructions in mindfulness practices (e.g. "…noticing any thoughts or urges to check your phone and seeing if you can just notice those thoughts or urges…")<br>7.5. Advise students when practicing mindfulness to turn their smart devices (e.g. phones, smartwatch) to silent mode or turn off the notifications.	12.3. Avoidance/reducing exposure to cues for the behaviour<br>[This recommendations apply to mindfulness in general. Therefore, no specific unwanted behaviours were targeted].

			12.1. Provide recommendations on how to find an appropriate place and time to practice mindfulness.<br>12.2. Advise students to restructure their social environment when they feel that it is a barrier to their mindfulness practice (e.g. being aware of their usual family gathering times, as it might not be a suitable time for practice).<br>12.5. see technology.	
Technology<br>• Good access to necessary equipment<br>• Varied experiences with network connectivity	Environmental context and resources.	3.2. Social support (practical)<br>7.1. Prompts/cues.<br>7.5. Remove aversive stimulus<br>12.1. Restructuring the physical environment.<br>12.2. Restructuring the social environment<br>12.5. Adding objects to the environment.	See environment for all the other BCTs.<br>12.5. Inform students about the necessary equipment to participate in the online MBI (e.g. a laptop/phone, network) so they can prepare for it in advance.<br>12.5. Provide the online MBI with offline functionality (e.g. recorded sessions, access to the course without necessarily a network)	See environment
		**Social opportunity**		
Influence of social environment on mindfulness practice.<br>• Lack of mindfulness practice among	Social influences<br><br>Subjective Norms	3.1. Social support (unspecified)<br>3.2. Social support (practical)<br>6.2. Social comparison<br>6.3. Information about others' approval<br>10.4. Social reward	3.1. Arrange for peer support from students with prior mindfulness experience to offer appropriate guidance to students with less experience.<br>3.2. Provide an information sheet that contains brief information about mindfulness and how others can support students during their practice. Students can also share this sheet with others.	NA

family and friends<br>• Impact of other's opinion on student's practice<br>• Variation in support from family and friends			6.2. Provide examples of university students who have practiced mindfulness and encourage students to reflect on their own practice.<br>6.3. Share information about other students' experiences of practicing mindfulness (e.g. whether they like it or not).<br>10.4. Provide positive social reinforcements to students as a reward for their mindfulness practice (e.g. encouraging messages such as 'Well done!').	
Societal norms and perspectives<br>• (lack of) awareness facilitating acceptance of mindfulness.<br>• Consistency of mindfulness with Arabic culture and Islam values	Norms<br><br>Subjective norms<br><br>Social learning/ imitation	6.1. Demonstration of the behaviour<br>6.2. Social comparison<br>6.3. Information about others' approval<br>9.1. Credible source	6.1. Provide videos or pictures demonstrating someone from Saudi or Arab culture practicing mindfulness.<br>6.2./ 6.3. See influence of social environment on mindfulness practice.<br>9.1. Provide examples of other Saudi students experiences' with mindfulness.<br>9.1. Provide examples of how other Saudi students perceive the relationship between mindfulness and their personal values. Encourage students to reflect on that as well.<br>9.1. Encourage academic staff or relevant figures to provide short talks about mindfulness for students at the university.	NA
		**Reflective motivation**		
Anticipated beliefs about the	Beliefs about the	5.1. Information about health consequences.	5.1. Provide information about the health benefits of mindfulness reported by university students.	5.5. Anticipated regret [This recommendation apply to mindfulness in general.

benefits of mindfulness<br>• Self and emotions discovery.<br>• Reduce psychologica l distress<br>• Improve psychologica l wellbeing and flexibility<br>• Improve responses to situations<br>• Acceptance and appreciation<br>• Improve cognitive skills.<br>• Awareness of the body and present moment.	consequenc es	5.2. Salience of consequences.<br>5.3. Information about social and environmental consequences.<br>5.6. Information about emotional consequences<br>9.2. Pros and cons<br>9.3. Comparative imagining of future outcomes<br>10.1. Material incentive (behaviour)<br>10.8. Incentive (outcome)<br>10.10. Reward (outcome)	5.2. Share a real-world example of Saudi students experiencing the benefits of mindfulness practice.<br>5.3. Provide information about the positive impact of mindfulness on students' social lives.<br>5.6. Provide information about the emotional benefits of mindfulness reported by university students.<br>9.2. Encourage students to reflect on the advantages and disadvantages of practicing mindfulness.<br>9.3. Highlight to students and encourage them to reflect on how learning mindfulness skills can improve their lives (e.g. by being present, noticing thoughts and feelings and making wiser choices), rather than falling into the usual habitual patterns.<br>10.1. Provide positive reinforcement (e.g. voucher) when completing the course.<br>10.8. Inform students that they will be receiving an incentive when completing the course.<br>10.10. Ensure the delivery of positive reinforcement (e.g. voucher) when students complete the course.	Therefore, no specific unwanted behaviours were targeted].
A range of experienced beliefs about the benefits of mindfulness	Beliefs about the consequenc es	See anticipated beliefs about the benefits of mindfulness.	See anticipated beliefs about the benefits of mindfulness.	See anticipated beliefs about the benefits of mindfulness.

Beliefs about the perceived side effects of mindfulness<br>• Negative impact on people experiencing psychological distress<br>• Annoying and boring	Beliefs about the consequences	5.1. Information about health consequences.	See anticipated beliefs about the benefits of mindfulness all the other BCTs.<br>5.1. Provide information and normalise common challenges of practicing mindfulness, especially those encounter in initial stages (e.g. feeling bored, mind wandering).	NA
Intentions to engage in mindfulness practice<br>• Mindfulness is needed in different situations<br>• Varying intentions to engage in online mindfulness courses.<br>Perceived facilitators for mindfulness practice	Intention	1.1. Goal setting (behaviour)<br>5.1. Information about health consequences<br>1.9. Commitment<br>10.8. Incentive (outcome)	1.1. Invite and encourage students to set weekly goals for their daily mindfulness practice.<br>5.1. Explain to the students that benefits of mindfulness come with consistent practice of mindfulness exercises over time.<br>1.9. Prompt students to share their commitment to practicing mindfulness with someone else (e.g. telling them), or to post about it on their social media.<br>10.8. See anticipated beliefs about the benefits of mindfulness	
		**Automatic motivation**		

A range of emotions associated with mindfulness practice	Emotion	11.2. Reduce negative emotions.	11.2. Validate and normalise negative emotions related to mindfulness practice (e.g. fear of failure).	NA
