# Supplementary material for: Facilitators, Barriers, and Cultural Appropriateness of Mindfulness-Based Interventions Among Saudi Female University Students: Qualitative Study
Source: JMIR Form Res. 2025 Dec 19;9:e78532. doi: 10.2196/78532 (PMC12716633; doi:10.2196/78532)
Supplement: Multimedia Appendix 5 [file formative-v9-e78532-s005.pdf]

Recommendations from the cultural adaptation framework by Bernal et al

Domain<br>• Themes	Recommendations
**Language**<br>• Variation in language preferences	• Deliver the intervention in both Arabic and English to accommodate students' preferences.<br>• In cases where resources are limited, prioritise delivering the intervention in Arabic, the native language of students.
**People**<br>• Varying preferences for the gender of instructors<br>• Preference of personalised written language	• The gender of the instructor delivering the audio-recording of mindfulness exercises is unrestricted. Both female and male voices are appropriate.<br>• Provide written content in the Arabic language in the singular and personalised form, emphasising direct communication towards the student (e.g. "السماح لأفكارك ومشاعرك…")
**Metaphor**<br>• Incorporating metaphors associated with Saudi and Arab culture<br>• Varied viewpoints in using Islamic-related metaphors	• Incorporate metaphors (e.g. sayings, poems, wisdom, examples) that resonate with Saudi and/or Arab culture and values.<br>• Incorporate a few metaphors related to the Islamic context.
**Content**<br>• Acceptability of mindfulness exercises<br>• Awareness facilitating acceptance of mindfulness exercises	• Mindfulness exercises in their typical form can be incorporated.<br>• Integrate mindfulness concepts and related exercises into Saudi culture by aligning them with local values and social habits.<br>• Integrate mindfulness concepts and related exercises into the environment of Saudi universities.<br>• Provide a brief introduction to the mindfulness exercises.<br>• Address and normalise common misconceptions and/or challenges alongside each exercise (e.g. during a body scan, explicitly clarify that the goal is not solely relaxation).
**Goals**<br>• Variation in students' goals for the online MBIs	Introduce mindfulness concepts and include exercises that align with students' goals. This includes:<br>• Provide psychoeducation, introduce mindfulness, and address common misconceptions throughout the intervention course to support students in understanding what mindfulness is.

	• Incorporate mindfulness concepts and exercises that support students in managing psychological distress and coping with stress (e.g. sitting mindfulness meditation: body scan, three-steps mindfulness breathing space).<br>• Incorporate mindfulness concepts and exercises that contribute to improving students' psychological well-being (e.g. compassion, kindness, and gratitude).<br>• Incorporate mindfulness concepts and exercises that aim to support students connect with their internal experiences (e.g. thoughts, emotions), and respond better to these experiences (e.g. understanding the rules of interpretations, bringing awareness to pleasant and unpleasant moments).<br>• Provide strategies and mindfulness exercises to support students in incorporating mindfulness into their daily lives (e.g. mindful routine activities).<br>• Provide strategies to support students in maintaining their mindfulness practice during and after completing the intervention.<br>• Share information with students about how practicing mindfulness can potentially benefit academic performance for university students (e.g. perceived benefits reported by other university students).
**Concept**<br>• Students' perspective on mindfulness aligned with its core principles<br>• Key factors facilitating mindfulness understanding	• Introduce mindfulness to students in its typical formulation (i.e. "paying attention in a practical way on purpose in the present moment and nonjudgmentally.")<br>• Clarify the presentation of mindfulness to students by:<br>− Provide a precise definition of mindfulness, along with its potential benefits.<br>− Clarify the meaning of "nonjudgmentally" in a simple manner.<br>− Illustrate concepts with real-world examples throughout the intervention to enhance understanding.<br>− Normalise common challenges beginners might face (e.g. mind wandering).
**Method**<br>• Variation in preferred online platforms<br>• Preferences for receiving reminders or prompts<br>• Varying intentions to engage in an online peer-support forum	• Offer students flexibility by providing the intervention via different online platforms, such as a website and a software application.<br>• Send reminders or prompts to practice mindfulness, with a low frequency (e.g. 3 times a week). Where applicable, allow students to personalise and control the frequency based on their needs.<br>• Provide a peer-support forum for students; however, this should be considered supplementary rather than an essential part of the intervention.<br>• Offer flexibility to students about the duration and intensity of the intervention by providing both a briefer version (≤4 weeks) and a longer version (>4 weeks) from which they can choose according to their needs.

• Varying preferences about the duration of the online MBI and mindfulness exercises	• Offer flexibility in the duration of mindfulness exercises by providing both a brief version (≤15 minutes) and a longer version (>15 minutes).<br>• Inform students about the time required to complete the intervention, sessions, and exercises.
**Context**<br>• Factors facilitating mindfulness implementation within Saudi society<br>• Factors facilitating mindfulness implementation within university setting	• Use media to raise awareness about mindfulness within Saudi society.<br>• Implement effective advertising strategies at the university to inform students about mindfulness and available courses. This might include using the university's social media, engaging academic staff to inform students during lectures, and conducting brief talks directly to students.<br>• Facilitate mindfulness practices for university students by offering dedicated time for mindfulness during lectures and providing access to such courses through the university.<br>• Encourage students' mindfulness practice through potential reinforcements offered by the university (e.g. receiving skill/training hours).
