# Supplementary material for: Facilitators, Barriers, and Cultural Appropriateness of Mindfulness-Based Interventions Among Saudi Female University Students: Qualitative Study
Source: JMIR Form Res. 2025 Dec 19;9:e78532. doi: 10.2196/78532 (PMC12716633; doi:10.2196/78532)
Supplement: Checklist 1 [file formative-v9-e78532-s006.pdf]

Consolidated Criteria for Reporting Qualitative Studies (COREQ): 32-item checklist.

No	Item	Guide questions/description	Reported on page No.
**Domain 1: Research team and reflexivity**			
Personal Characteristics			
1.	Interviewer/facilitator	Which author/s conducted the interview or focus group?	P. 4
2.	Credentials	What were the researcher's credentials? *E.g. PhD, MD*	P. 5, 6
3.	Occupation	What was their occupation at the time of the study?	P. 5, 6
4.	Gender	Was the researcher male or female?	P. 5
5.	Experience and training	What experience or training did the researcher have?	P. 5, 6
Relationship with participants			
6.	Relationship established	Was a relationship established prior to study commencement?	P. 6
7.	Participant knowledge of the interviewer	What did the participants know about the researcher? e.g. *personal goals, reasons for doing the research*	P. 6
8.	Interviewer characteristics	What characteristics were reported about the interviewer/facilitator? e.g. *Bias, assumptions, reasons and interests in the research topic*	P. 6
**Domain 2 : Study design**			
Theoretical framework			
9.	Methodological orientation and Theory	What methodological orientation was stated to underpin the study? *e.g. grounded theory, discourse analysis, ethnography, phenomenology, content analysis*	P. 4, 5
Participant selection			
10.	Sampling	How were participants selected? *e.g. purposive, convenience, consecutive, snowball*	P. 3, 4
11.	Method of approach	How were participants approached? e.g. *face-to-face, telephone, mail, email*	P. 4
12.	Sample size	How many participants were in the study?	P. 6
13.	Non-participation	How many people refused to participate or dropped out? Reasons?	P. 6
Setting			
14.	Setting of data collection	Where was the data collected? e.g. *home, clinic, workplace*	P. 4, 6
15.	Presence of non-participants	Was anyone else present besides the participants and researchers?	P. 4, 6
16.	Description of sample	What are the important characteristics of the sample? *e.g. demographic data, date*	P. 6, Table 1
Data collection			
17.	Interview guide	Were questions, prompts, guides provided by the authors? Was it pilot tested?	P. 3, Appendix 1
18.	Repeat interviews	Were repeat interviews carried out? If yes, how many?	NA
19.	Audio/visual recording	Did the research use audio or visual recording to collect the data?	P. 4
20.	Field notes	Were field notes made during and/or after the interview or focus group?	P. 4
21.	Duration	What was the duration of the interviews or focus group?	P. 6
22.	Data saturation	Was data saturation discussed?	P. 4

23.	Transcripts returned	Were transcripts returned to participants for comment and/or correction?	P. 13
**Domain 3: Analysis and findings**			
Data analysis			
24.	Number of data coders	How many data coders coded the data?	P. 4, 5
25.	Description of the coding tree	Did authors provide a description of the coding tree?	P. 4, 5
26.	Derivation of themes	Were themes identified in advance or derived from the data?	P. 4, 5
27.	Software	What software, if applicable, was used to manage the data?	P. 4, 5
28.	Participant checking	Did participants provide feedback on the findings?	P. 13
Reporting			
29.	Quotations presented	Were participant quotations presented to illustrate the themes / findings? Was each quotation identified? e.*g. participant number*	Table 2, 3, Appendix 2, 3
30.	Data and findings consistent	Was there consistency between the data presented and the findings?	P. 7-11, Tables 2, 3
31.	Clarity of major themes	Were major themes clearly presented in the findings?	P. 7-11, Table 2, 3
32.	Clarity of minor themes	Is there a description of diverse cases or discussion of minor themes?	P. 7-11, Table 2, 3, Appendix 2, 3

Note: Adapted from *"Consolidated criteria for reporting qualitative research (COREQ): A 32-item checklist for interviews and focus groups" by A. Tong, P. Sainsbury, & J. Craig, 2007, International Journal for Quality in Health Care, 19(6), 349–357.*
